# Synthesis of Xanthones, Thioxanthones and Acridones by a Metal-Free Photocatalytic Oxidation Using Visible Light and Molecular Oxygen

**DOI:** 10.3390/molecules26040974

**Published:** 2021-02-12

**Authors:** Alejandro Torregrosa-Chinillach, Rafael Chinchilla

**Affiliations:** Department of Organic Chemistry, Faculty of Sciences, and Institute of Organic Synthesis (ISO), University of Alicante, Apdo. 99, 03080 Alicante, Spain; alex.torregrosa@ua.es

**Keywords:** photo-oxidation, photocatalysis, xanthones, thioxanthones, acridones

## Abstract

9*H*-Xanthenes, 9*H*-thioxanthenes and 9,10-dihydroacridines can be easily oxidized to the corresponding xanthones, thioxanthones and acridones, respectively, by a simple photo-oxidation procedure carried out using molecular oxygen as oxidant under the irradiation of visible blue light and in the presence of riboflavin tetraacetate as a metal-free photocatalyst. The obtained yields are high or quantitative.

## 1. Introduction

In the last years, photocatalysis has emerged enormously as a powerful tool in synthetic organic chemistry when promoting electron transfer-involved chemical transformations [1,2,3,4,5]. In this context, the use of visible light results particularly attractive due to its lack of absorbance by organic compounds [6,7]. This reduces side reactions, frequently associated with classical photochemical reactions carried out with high energy UV light absorbed directly by the molecule. When less energetic and innocuous visible light is employed, the addition of catalytic amounts of a sensitizer as photocatalyst is required, but the reaction setup results simple and is not too different from that of typical thermal chemistry, except for the light source. Among the most frequent and successful transformations carried out under these conditions are photoredox reactions [8,9,10,11,12,13,14,15,16,17].

The visible light-induced photocatalytic oxidation of benzylic bonds [18,19], especially when the final oxidant is the cheap and environmentally friendly molecular oxygen [20,21], results in a convenient and direct technique to generate the corresponding carbonyl systems. This concept can be applied to the preparation of interesting structural compounds such as xanthones, thioxanthones and acridones by oxidation of benzylic C-H bonds of appropriate precursors. Thus, xanthones (9*H*-xanthen-9-ones) are interesting to prepare [22,23], as their structural motif is present in compounds showing a multitude of biological activities, including α-glucosidase inhibition, as well as anticancer, anti-Alzheimer and anti-inflammatory properties [24,25,26,27]. Besides, thioxanthones have also shown interesting antitumor activities [28]. Moreover, acridones [acridin-9(10*H*)-ones] are important moieties present in antiviral and antitumor agents [29,30,31].

Xanthone has been obtained by photocatalytic oxidation of the benzylic methylene position in 9*H*-xanthene. Thus, ruthenium complexes as catalysts have been used, the oxidant being oxygen [32] or iodate salts [33,34]. Iron complexes have also been employed as catalysts under aerobic conditions [35,36,37], as well as a copper complex [38] or copper(II) chloride [39]. All these procedures, although effective, employ a metal-containing catalyst, something not too environmentally desirable. Therefore, procedures involving metal-free conditions have been explored. Thus, functionalized carbon-dots have been employed as catalysts for the photo-oxidation of 9*H*-xanthene, using *tert*-butyl hydroperoxide as oxidant [40], as well as graphitic carbon nitride, oxygen being the oxidant in this case [41]. Xanthones have also been prepared by visible-light aerobic oxidation of different 9*H*-xanthenes, a reaction promoted by the combination of 2,3-dichloro-5,6-dicyano-1,4-benzoquinone (DDQ), *tert*-butyl nitrite and acetic acid [42]. In addition, 9*H*-xanthene has been photo-oxidized to xanthone using 9-mesityl-10-methylacridinium tetrafluoroborate dye as catalyst and Selectfluor as oxidant [43]. Moreover, a flavin-derivative has been shown to photocatalyze this oxidation reaction under aerobic conditions, although in the presence of trifluoroacetic acid [44].

The photocatalytic oxidation of the methylene position in *N*-substituted 9,10-dihydroacridines (acridanes) leading to acridones has been less frequently explored. Thus, irradiation of *N*-methylacridane in the presence of a manganese complex and oxygen gave the corresponding *N*-methylacridone [45], a transformation which has also been carried out with air but using a ruthenium complex as photocatalyst [46]. In addition, the former combination of DDQ, *tert*-butyl nitrite and acetic acid has also been used for the visible light-induced oxidation of 9,10-dihydroacridines to acridones [42].

We report now a very simple and environmentally benign preparation of xanthones, thioxanthones and acridones carried out by irradiation of 9*H*-xanthenes, 9*H*-thioxanthenes and 9,10-dihydroacridines, respectively, with visible blue light in the presence of oxygen and using a low-costing metal-free photocatalyst.

## 2. Results and Discussion

**Figure 1:**
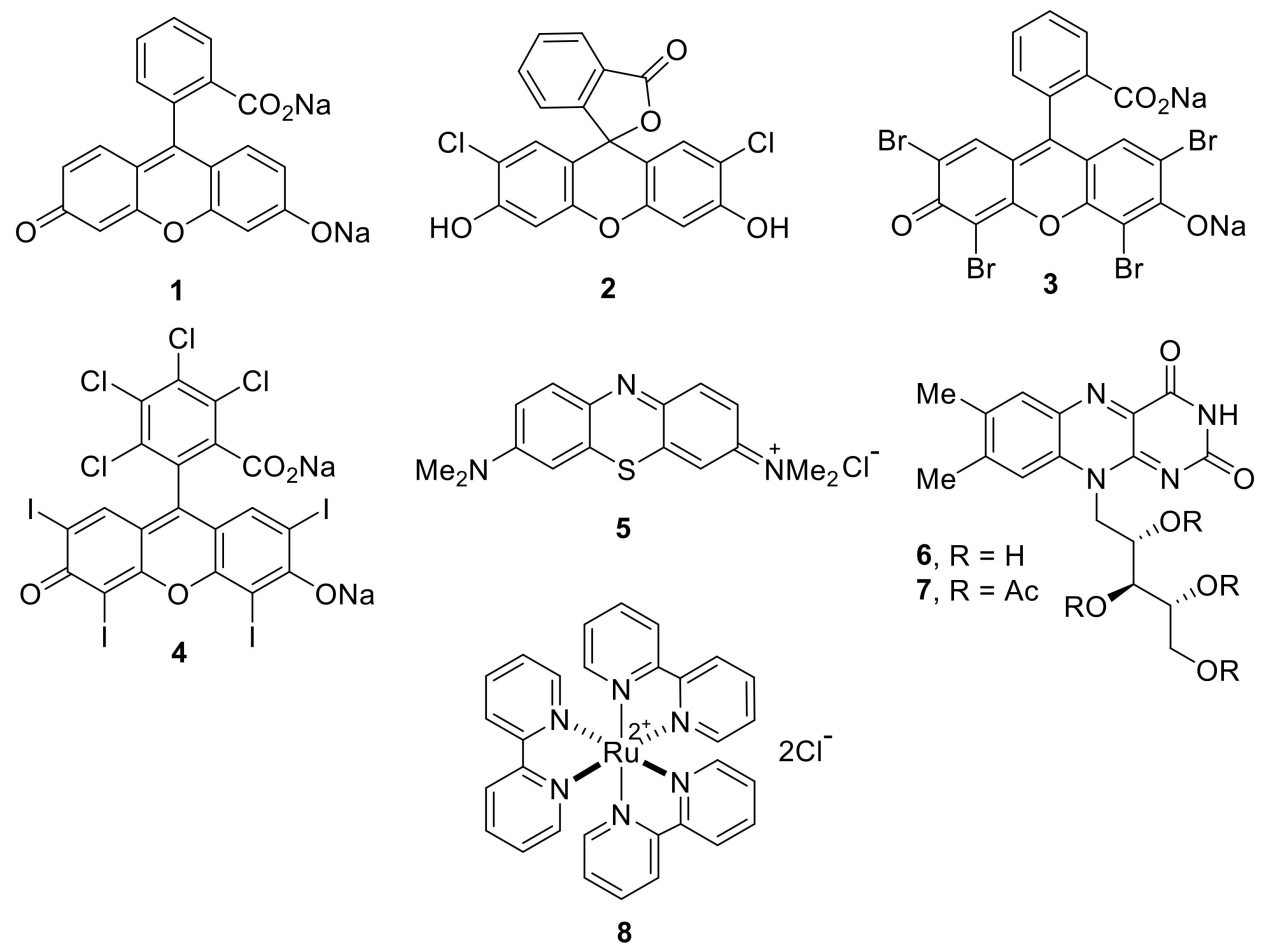
Photocatalysts employed in this study.

Different organic dyes **1**–**7** (Figure 1), employed previously in other photoredox reactions [13,47,48], were explored as photocatalysts in the model photo-oxidation of 9*H*-xanthene (**9a**) to xanthone (**10a**), under a molecular oxygen atmosphere (1 atm, balloon) and irradiation with visible blue light during 8 h (Table 1).

When fluorescein sodium salt (**1**) was used as photocatalyst (5 mol%) in acetonitrile as solvent, the desired oxidation product **10a** was obtained, although in a low conversion (Table 1, entry 1). Moreover, the proton nuclear magnetic resonance (^1^H NMR) analysis of the reaction crude also revealed the presence of a small amount of bixanthene **11** as an undesirable coupling byproduct (H9 singlet at 4.18 ppm [49]) (Table 1, entry 1). Changing the photocatalyst to 2′,7′-dichlorofluorescein (**2**) increased noticeably the conversion to xanthone **10a**, with no bixanthene **11** being observed (Table 1, entry 2). The conversion to xanthone **10a** resulted furtherly increased to 74% using eosin Y sodium salt (**3**) as photocatalyst, although, again, the presence of the bixanthene byproduct **11** was detected (Table 1, entry 3). However, the reaction performed poorly when photocatalyzed by rose bengal sodium salt (**4**) (Table 1, entry 4), similarly to when methylene blue (**5**) was employed (Table 1, entry 5).

Riboflavin (**6**), also known as vitamin B2, is a cheap naturally occurring yellow compound, and hence it can absorb visible light with a maximum in the blue range, increasing its redox power upon excitation [50,51,52,53,54]. Riboflavin performed poorly under the former reaction conditions (Table 1, entry 6), but the use of riboflavin tetraacetate (**7**) as photocatalyst gave full conversion to the desired xanthone **10a**, with no traces of bixanthene **11** being observed in ^1^H NMR (Table 1, entry 7). Perhaps the higher solubility of **7** in organic solvents plays a role in its activity compared to **6**. Moreover, we also explored if a metal-containing photocatalyst could be used, employing the very known visible light-absorbing complex Ru(bpy)_3_Cl_2_ (bpy = 2,2′-bipyridyl) (**8**) (Figure 1) [12,55,56], although its performance resulted very poor under these conditions (Table 1, entry 8).

Then, we explored the use of other solvents using riboflavin tetraacetate (**7**) as the optimum photocatalyst. Thus, the use of *N*,*N*’-dimethylformamide (DMF) gave a similar mixture of xanthone **10a** and the bixanthene byproduct **11** (Table 1, entry 9), whereas the use of methanol gave both compounds in lower conversions (Table 1, entry 10). In addition, when dichloromethane was used as solvent, a mixture of **10a** and **11** was obtained (Table 1, entry 11), but, when another chlorinated solvent such as 1,2-dichloroethane was used, the starting xanthene **9a** was fully converted to the oxidation product **10a** (Table 1, entry 12). With these results in hand, we chose acetonitrile as the solvent, attempting to avoid the use of a chlorinated solvent.

We also carried out the reaction under air in an open flask using the best reaction conditions (**7** as photocatalyst and MeCN as solvent), but a conversion of only 55% to **10a** was achieved with a noticeable 24% conversion to bixanthene **11** (Table 1, entry 13). In addition, the reaction was carried out after just sparging molecular oxygen into the solution and closing the reaction flask, avoiding the use of the balloon, but a 73% conversion to **10a** and 26% for **11** was detected (Table 1, entry 14). Moreover, the reaction was carried out in the absence of any light, giving almost no reaction (Table 1, entry 15). Finally, the oxidation was attempted in the absence of photocatalyst **7**, with only 8% conversion to **10a** being obtained (Table 1, entry 16). We also used a lower photocatalyst loading (2 mol%), but a remaining 22% of starting **9a** was observed.

**Figure 2:**
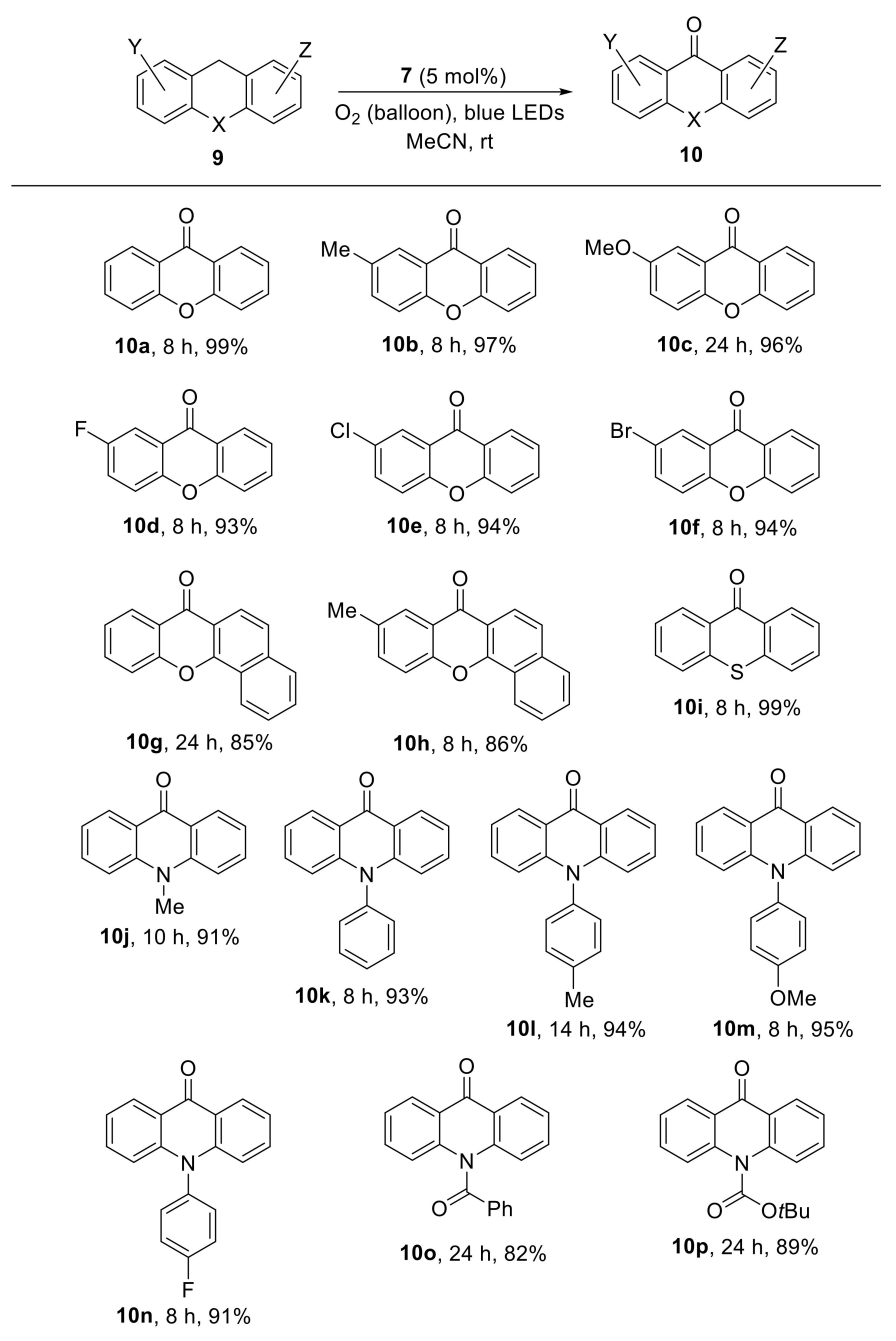
Photocatalyzed oxidation of 9*H*-xanthenes, 9*H*-thioxanthene and 9,10-dihydroacridines. Reaction time and yield, after isolation by flash chromatography, detailed for each compound.

Once we found the most appropriate reaction conditions for the photo-oxidation reaction, we extended the procedure to other 9*H*-xanthenes and related systems, observing in all cases full conversion to the desired oxidated derivative (Figure 2). Thus, when the methyl- or methoxy-substituted 9*H*-xanthenes **9b** and **9c** were photo-oxidized, the corresponding xanthones **10b** and **10c** were obtained in high isolated yields (Figure 2). In addition, when 9*H*-xanthenes with substituents such as fluoro, chloro or bromo were employed in the reaction, the corresponding halogenated xanthones **10d**, **10e** and **10f**, respectively, were also isolated in high yields (Figure 2). Moreover, benzocondensed 9*H*-xanthenes were also employed in the oxidation reaction, affording the corresponding xanthones **10g** and **10h** (Figure 2). Furthermore, 9*H*-thioxanthene was also explored for the photo-oxidation reaction, affording quantitatively the final thioxanthone **10i** (Figure 2).

We also used this photo-oxidation reaction with *N*-substituted 9,10-dihydroacridines (acridanes), obtaining excellent results of the corresponding acridones (Figure 2). Thus, when *N*-methyl-9,10-dihydroacridine was employed, acridone **10j** was isolated in a 91% yield, whereas *N*-phenyl-9,10-dihydroacridine afforded the oxidation derivative **10k** in 93% yield. In addition, the presence of different substituents on the *N*-phenyl ring in the last acridane, such as methyl, methoxy or fluoro, allowed isolating the corresponding acridones **10l**, **10m** and **10n**, respectively, in high yields. Moreover, when a benzoyl *N*-substituent was present in the starting 9,10-dihydroacridine, the *N*-benzoylated acridone **10o** was isolated in 82% yield. Furthermore, the presence of an acid-sensitive *N*-substituent, such as the *tert*-butoxycarbonyl (Boc) group was tolerated under these neutral oxidation conditions, allowing to isolate the *N*-Boc-acridone **10p** in 89% yield.

This photo-oxidation reaction probably takes place similarly to other proposed aerobic photocatalytic oxidation of benzylic systems [57], although particularized to the use of riboflavin as photocatalyst [44]. Thus, it is known that the riboflavin nucleus is excited in the presence of visible light [50,51,52,53,54] and then would be able to oxidize the benzyl system to a cation radical, generating a riboflavin radical anion after a single-electron transfer (SET) process. This riboflavin radical anion can convert oxygen to a superoxide radical anion which reduces the benzyl cation radical to a benzyl radical able to react with oxygen to generate a benzyl hydroperoxide which would decompose to the carbonyl system. Singlet oxygen is probably involved in this process, as it is known that it can be produced by flavin sensitization [58].

**Scheme 1:**
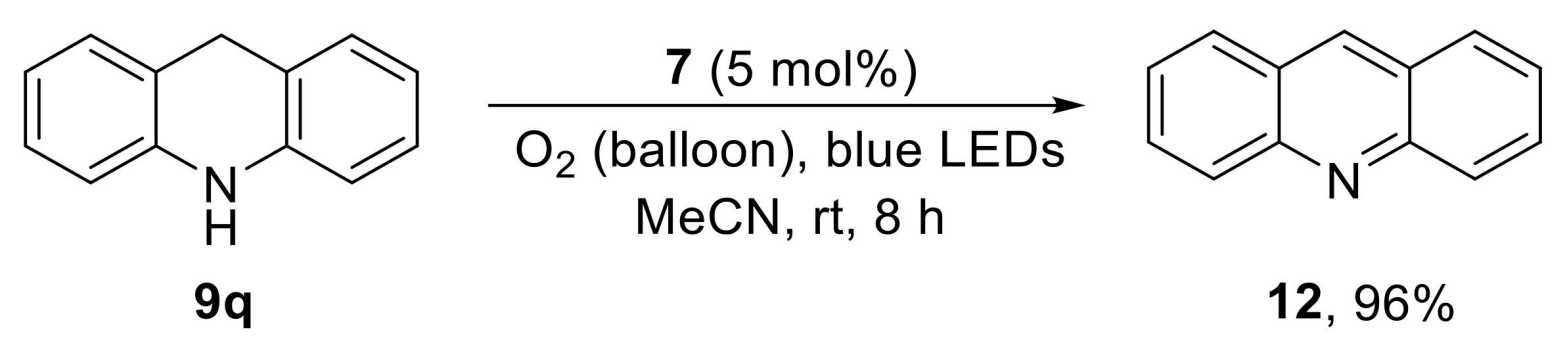
Photo-oxidation of 9,10-dihydroacridine.

We also explored this photo-oxidation reaction using the simple *N*-unsubstituted 9,10-dihydroacridine **9q** as starting material, but, instead of the corresponding *N*-unsubstituted acridone, fully aromatized acridine (**12**) was isolated after 8 h in a 96% yield (Scheme 1). This photocatalytic oxidative dehydrogenation of acridine has been reported previously [42] and used as a procedure for the aromatization of *N*-heterocycles [59,60].

## 3. Materials and Methods

### 3.1. General Information

All reagents and solvents employed were of the best grade available and were used without further purification. Riboflavin tetraacetate (**7**) was obtained by acetylation of riboflavin (**6**) as described [61]. Substituted 9*H*-xanthenes **9b**–**h** were prepared as reported [62]. Starting dihydroacridines **9j** [63], **9k**–**n** [64], **9o** [65] and **9p** [66] were obtained by *N*-substitution of 9,10-dihydroacridine (**9q**) following literature procedures. 9,10-Dihydroacridine (**9q**) was prepared by reduction of acridine as described [67]. The ^1^H and ^13^C spectra were recorded at room temperature on a Bruker Oxford AV300 at 300 MHz and 75 MHz, on a Bruker Oxford AV400 at 400 MHz and 101 MHz, or a Bruker Avance DRX500 at 500 MHz and 125 MHz, respectively (Bruker, Billerica, MA, USA), using CDCl_3_ as solvent and TMS as the internal standard. Reactions were carried out in borosilicate vials (length: 4 cm, diameter: 1.4 cm, volume: 4 mL) and irradiated in a Evoluchem^TM^ PhotoRedOx Box Duo (HepatoChem Inc., Beverly, MA, USA) equipped with two Evoluchem^TM^ CREE XPE 450–455 nm 18W LEDs (HCK1012-01-002) (HepatoChem Inc., Beverly, MA, USA).

### 3.2. General Procedure for the Photocatalytic Oxidation Reaction.

A mixture of **9** (0.2 mmol) and **7** (0.01 mmol, 5.4 mg) was dissolved in acetonitrile (0.4 mL), and the solution was bubbled with O_2_ for 2 min. The flask was closed and stirred at room temperature under an O_2_ atmosphere (balloon) with irradiation by blue LEDs during the indicated reaction time (Figure 2). After that, H_2_O (2 mL) was added, and the reaction product was extracted with AcOEt (3 × 2 mL). The combined organic phases were dried over MgSO_4_, filtered and evaporated under reduced pressure (15 Torr) to get the crude product, which was purified by flash column chromatography on silica gel (*n*-hexane/AcOEt gradients). The adducts **10** and **12** were identified by comparison of their NMR data with those of the literature (Supplementary Materials, NMR spectra).

*9*H*-Xanthen-9-one* (**10a**) [68]. White solid (39 mg, 99%); ^1^H NMR (400 MHz): δ_H_ = 8.34 (dd, *J* = 8.0, 1.5 Hz, 2H), 7.76–7.67 (m, 2H), 7.48 (d, *J* = 8.6 Hz, 2H), 7.41–7.33 (m, 2H) ppm; ^13^C NMR (101 MHz): δ_C_ = 177.3, 156.3, 134.9, 126.8, 124.0, 122.0, 118.1 ppm.

*2-Methyl-9*H*-xanthen-9-one* (**10b**) [68]. White solid (41 mg, 97%); ^1^H NMR (300 MHz): δ_H_ = 8.34 (dd, *J* = 8.0, 1.7 Hz, 1H), 8.16–8.08 (m, 1H), 7.75–7.66 (m, 1H), 7.56–7.44 (m, 2H), 7.42–7.32 (m, 2H), 2.47 (s, 3H) ppm; ^13^C NMR (101 MHz): δ_C_ = 177.4, 156.3, 154.5, 136.2, 134.7, 133.8, 126.8, 126.1, 123.8, 121.9, 121.6, 118.1, 117.9, 21.0 ppm.

*2-Methoxy-9*H*-xanthen-9-one* (**10c**) [68]. White solid (43 mg, 96%); ^1^H NMR (300 MHz): δ_H_ = 8.35 (dd, *J* = 8.0, 1.6 Hz, 1H), 7.76–7.67 (m, 2H), 7.52–7.29 (m, 4H), 3.92 (s, 3H) ppm; ^13^C NMR (75 MHz): δ_C_ = 177.2, 156.2, 156.1, 151.1, 134.7, 126.8, 125.0, 123.8, 122.2, 121.4, 119.5, 118.1, 105.9, 56.1 ppm.

*2-Fluoro-9*H*-xanthen-9-one* (**10d**) [68]. White solid (40 mg, 93%); ^1^H NMR (300 MHz): δ_H_ = 8.33 (dd, *J* = 8.0, 1.6 Hz, 1H), 8.02–7.93 (m, 1H), 7.80–7.70 (m, 1H), 7.55–7.35 (m, 4H) ppm; ^13^C NMR (101 MHz): δ_C_ = 176.7 (d, *J* = 2.2 Hz), 158.9 (d, *J* = 245.3 Hz), 156.3, 152.5 (d, *J* = 1.6 Hz), 135.2, 126.8, 124.3, 123.1 (d, *J* = 25.3 Hz), 122.8 (d, *J* = 7.1 Hz), 121.2, 120.1 (d, *J* = 7.8 Hz), 118.1, 111.6 (d, *J* = 23.5 Hz) ppm.

*2-Chloro-9*H*-xanthen-9-one* (**10e**) [69]. White solid (43 mg, 94%); ^1^H NMR (300 MHz): δ_H_ = 8.40–8.23 (m, 2H), 7.81–7.61 (m, 2H), 7.55–7.33 (m, 3H) ppm; ^13^C NMR (75 MHz): δ_C_ = 176.3, 156.2, 154.6, 135.3, 135.1, 129.9, 126.9, 126.2, 124.4, 122.8, 121.6, 119.9, 118.2 ppm.

*2-Bromo-9*H*-xanthen-9-one* (**10f**) [68]. White solid (52 mg, 94%); ^1^H NMR (400 MHz): δ_H_ = 8.45–8.41 (m, 1H), 8.31 (dd, *J* = 8.0, 1.8 Hz, 1H), 7.82–7.70 (m, 2H), 7.51–7.45 (m, 1H), 7.43–7.35 (m, 2H) ppm; ^13^C NMR (101 MHz): δ_C_ = 176.1, 156.1, 155.0, 137.8, 135.3, 129.3, 126.9, 124.4, 123.2, 121.6, 120.1, 118.2, 117.2 ppm.

*7*H*-Benzo[*c*]xanthen-7-one* (**10g**) [68]. White solid (42 mg, 85%); ^1^H NMR (300 MHz): δ_H_ = 8.70–8.63 (m, 1H), 8.44–8.37 (m, 1H), 8.27 (d, *J* = 8.7 Hz, 1H), 7.95–7.89 (m, 1H), 7.81–7.64 (m, 5H), 7.48–7.41 (m, 1H) ppm; ^13^C NMR (101 MHz): δ_C_ = 177.0, 155.9, 153.8, 136.7, 134.5, 129.7, 128.2, 127.0, 126.7, 124.6, 124.2, 124.2, 123.0, 122.6, 121.6, 118.2, 117.7 ppm.

*9-Methyl-7*H*-benzo[*c*]xanthen-7-one* (**10h**) [70]. White solid (45 mg, 86%); ^1^H NMR (300 MHz): δ_H_ = 8.68–8.60 (m, 1H), 8.26 (d, *J* = 8.8 Hz, 1H), 8.19–8.14 (m, 1H), 7.95–7.87 (m, 1H), 7.75–7.64 (m, 3H), 7.56 (d, *J* = 1.3 Hz, 2H), 2.49 (s, 3H) ppm; ^13^C NMR (101 MHz): δ_C_ = 177.1, 154.1, 153.8, 136.6, 135.7, 134.4, 129.6, 128.2, 126.9, 126.0, 124.3, 123.9, 123.0, 122.2, 121.7, 117.9, 117.7, 21.1 ppm.

*9*H*-Thioxanthen-9-one* (**10i**) [68]. White solid (42 mg, 99%); ^1^H NMR (300 MHz): δ_H_ = 8.63 (ddd, *J* = 8.1, 1.5, 0.6 Hz, 2H), 7.67–7.56 (m, 4H), 7.53–7.46 (m, 2H) ppm; ^13^C NMR (101 MHz): δ_C_ = 180.1, 137.4, 132.4, 130.0, 129.4, 126.5, 126.1 ppm.

*10-Methylacridin-9(10*H*)-one* (**10j**) [68]. Yellow solid (38 mg, 91%); ^1^H NMR (300 MHz): δ_H_ = 8.55 (dd, *J* = 8.0, 1.6 Hz, 2H), 7.75–7.65 (m, 2H), 7.53–7.46 (m, 2H), 7.31–7.24 (m, 2H), 3.87 (s, 3H) ppm; ^13^C NMR (101 MHz): δ_C_ = 178.1, 142.7, 134.1, 128.0, 122.5, 121.5, 114.9, 33.9 ppm.

*10-Phenylacridin-9(10*H*)-one* (**10k**) [71]. Yellow solid (50 mg, 93%); ^1^H NMR (500 MHz): δ_H_ = 8.60 (dd, *J* = 8.0, 1.6 Hz, 2H), 7.74–7.69 (m, 2H), 7.68–7.64 (m, 1H), 7.53–7.48 (m, 2H), 7.40–7.36 (m, 2H), 7.31–7.27 (m, 2H), 6.76 (d, *J* = 8.6 Hz, 2H) ppm; ^13^C NMR (125 MHz): δ_C_ = 178.3, 143.3, 139.1, 133.4, 131.2, 130.2, 129.8, 127.4, 121.9, 121.7, 116.9 ppm.

*10-(*p*-Tolyl) acridin-9(10*H*)-one* (**10l**) [71]. Yellow solid (53 mg, 94%); ^1^H NMR (500 MHz): δ_H_ = 8.59 (dd, *J* = 8.0, 1.5 Hz, 2H), 7.52–7.47 (m, 4H), 7.29–7.23 (m, 4H), 6.80 (d, *J* = 8.6 Hz, 2H), 2.54 (s, 3H) ppm; ^13^C NMR (125 MHz): δ_C_ = 178.3, 143.5, 139.8, 136.5, 133.3, 131.8, 129.9, 127.4, 122.0, 121.6, 117.0, 21.5 ppm.

*10-(4-Methoxyphenyl) acridin-9(10*H*)-one* (**10m**) [71]. Yellow solid (57 mg, 95%); ^1^H NMR (300 MHz): δ_H_ = 8.58 (dd, *J* = 8.1, 1.4 Hz, 2H), 7.55–7.46 (m, 2H), 7.31–7.24 (m, 4H), 7.22–7.16 (m, 2H), 6.82 (d, *J* = 8.6 Hz, 2H), 3.95 (s, 3H) ppm; ^13^C NMR (125 MHz): δ_C_ = 178.3, 160.3, 143.7, 133.4, 131.6, 131.2, 127.4, 122.1, 121.6, 117.0, 116.3, 55.8 ppm.

*10-(4-Fluorophenyl) acridin-9(10*H*)-one* (**10n**) [71]. Yellow solid (52 mg, 91%); ^1^H NMR (300 MHz): δ_H_ = 8.59 (dd, *J* = 8.0, 1.7 Hz, 2H), 7.56–7.48 (m, 2H), 7.45–7.34 (m, 4H), 7.33–7.26 (m, 2H), 6.75 (d, *J* = 8.4 Hz, 2H) ppm; ^13^C NMR (101 MHz): δ_C_ = 178.3, 163.0 (d, *J* = 250.4 Hz), 143.3, 135.0 (d, *J* = 3.4 Hz), 133.6, 132.1 (d, *J* = 8.7 Hz), 127.6, 122.0, 121.9, 118.4 (d, *J* = 22.8 Hz), 116.7 ppm.

*10-Benzoylacridin-9(10*H*)-one* (**10o**) [65]. Yellow solid (49 mg, 82%); ^1^H NMR (500 MHz): δ_H_ = 8.57 (dd, *J* = 8.1, 1.3 Hz, 2H), 7.85–7.80 (m, 2H), 7.69–7.63 (m, 1H), 7.58–7.53 (m, 2H), 7.48–7.43 (m, 2H), 7.36–7.31 (m, 2H), 7.19 (d, *J* = 8.5 Hz, 2H) ppm; ^13^C NMR (101 MHz): δ_C_ = 178.2, 172.7, 139.9, 135.7, 133.9, 132.4, 131.5, 129.8, 127.9, 122.9, 121.7, 116.6 ppm.

Tert*-butyl 9-oxoacridine-10(9*H*)-carboxylate* (**10p**) [66]. Yellow solid (52 mg, 89%); ^1^H NMR (300 MHz): δ_H_ = 8.48–8.41 (m, 2H), 7.71–7.63 (m, 2H), 7.61–7.55 (m, 2H), 7.39–7.31 (m, 2H), 1.69 (s, 9H) ppm; ^13^C NMR (75 MHz): δ_C_ = 178.7, 152.0, 139.6, 133.5, 127.4, 123.4, 123.0, 117.7, 86.4, 27.9 ppm.

*Acridine* (**12**) [72]. Yellow solid (34 mg, 96%); ^1^H NMR (300 MHz): δ_H_ = 8.76 (s, 1H), 8.27 (d, *J* = 8.8 Hz, 2H), 7.99 (d, *J* = 8.5 Hz, 2H), 7.83–7.74 (m, 2H), 7.57–7.49 (m, 2H) ppm; ^13^C NMR (101 MHz): δ_C_ = 148.3, 137.2, 131.1, 128.7, 128.4, 126.6, 126.1 ppm.

## 4. Conclusions

Riboflavin tetraacetate acts as a convenient photocatalyst in the visible light-promoted benzylic oxidation of 9*H*-xanthenes, 9*H*-thioxanthenes and 9,10-dihydroacridines to xanthones, thioxanthones and acridones, respectively, using molecular oxygen as the oxidant in acetonitrile as solvent. This easy oxidation procedure allows the isolation of these interesting products in high to quantitative yields, using a methodology that avoids the use of metal-containing catalysts or chemical oxidants and working under neutral conditions.

## Figures and Tables

**Table 1:**
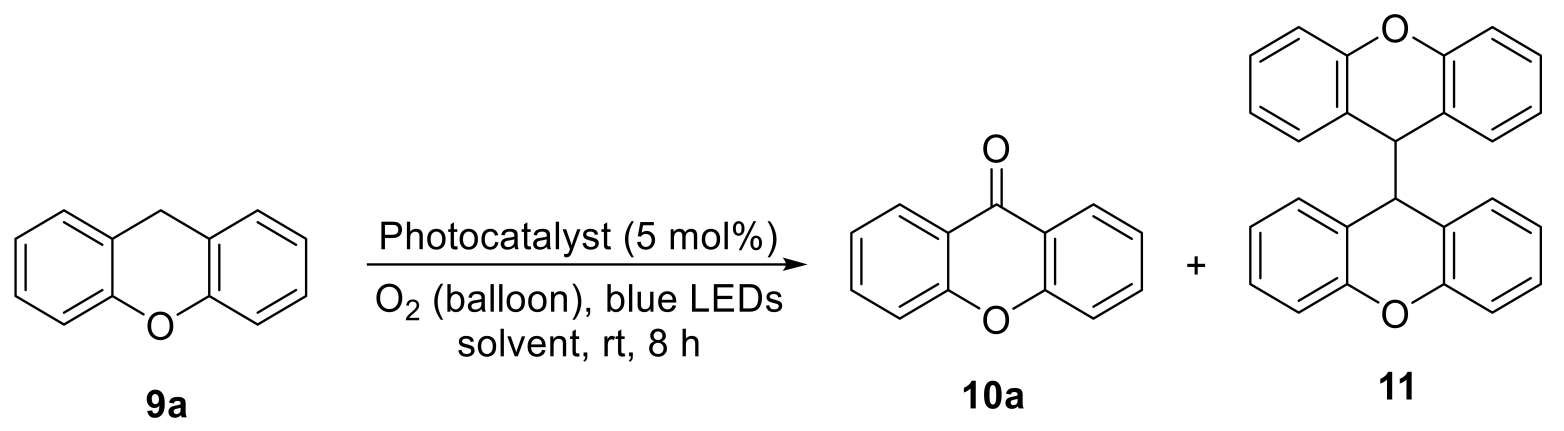
Screening and optimization of the reaction conditions for the photo-oxidation of 9*H*-xanthene.

Entry	Photocatalyst	Solvent	10a Conversion (%) ^a^	11 Conversion (%) ^a^
1	**1**	MeCN	13	4
2	**2**	MeCN	65	0
3	**3**	MeCN	74	15
4	**4**	MeCN	7	2
5	**5**	MeCN	10	0
6	**6**	MeCN	11	3
7	**7**	MeCN	100	0
8	**8**	MeCN	6	0
9	**7**	DMF	47	53
10	**7**	MeOH	31	13
11	**7**	CH_2_Cl_2_	56	44
12	**7**	ClCH_2_CH_2_Cl	100	0
13 ^b^	**7**	MeCN	55	24
14 ^c^	**7**	MeCN	73	26
15 ^d^	**7**	MeCN	2	0
16	-	MeCN	8	0

^a^ Relative to the starting material and determined by proton nuclear magnetic resonance (^1^H NMR) (300 MHz). ^b^ The reaction was carried out under air in an open flask. ^c^ The reaction was carried out without oxygen balloon, after just sparging the solution and closing the reaction flask. ^d^ The reaction was performed in the absence of light.

## Data Availability

The data presented in this study are available in insert article or supplementary material here.

## Supplementary Materials

The following are available online, Figures S1–S34 NMR spectra, Figure S35 spectrum of the LED lamp, and Figures S36–S43 UV–visible absorption spectra of the photocatalysts.

## Sample Availability

Samples of the compounds **10a**–**j** and **10m**–**p** are available from the authors.

## References

[1] Schroll P., König B. (2013). Early Pioneers of Organic Photochemistry. Chemical Photocatalysis.

[2] Ameta R., Ameta S.C. (2016). Photocatalysis: Principles and Applications.

[3] König B. (2017). Photocatalysis in organic synthesis-Past, present, and future. Eur. J. Org. Chem..

[4] Sideri I.K., Voutyritsa E., Kokotos C.G. (2018). Photoorganocatalysis, small organic molecules and light for organic synthesis: The awakening of a sleeping giant. Org. Biomol. Chem..

[5] Zhang J., Tian B., Wang L., Xing M., Lei J. (2018). Photocatalysis: Fundamentals, Materials and Applications.

[6] Liu Q., Wu L.-Z. (2017). Recent advances in visible-light-driven organic reactions. Natl. Sci. Rev..

[7] Stephenson C.R.J., Yoon T.P., MacMillan D.W.C., Stephenson C.R.J., Yoon T.P., MacMillan D.W.C. (2018). Visible Light Photocatalysis in Organic Chemistry.

[8] Narayanam J.M.R., Stephenson C.R.J. (2011). Visible light photoredox catalysis: Applications in organic synthesis. Chem. Soc. Rev..

[9] Tucker J.W., Stephenson C.R.J. (2012). Shining light on photoredox catalysis: Theory and synthetic applications. J. Org. Chem..

[10] Xuan J., Xiao W.-J. (2012). Visible-light photoredox catalysis. Angew. Chem. Int. Ed..

[11] Gambarotti C., Melone L., Caronna T., Punta C. (2013). O_2_-mediated photocatalytic functionalization of organic compounds: Recent advances towards greener synthetic routes. Curr. Org. Chem..

[12] Prier C.K., Rankic D.A., MacMillan D.W.C. (2013). Visible light photoredox catalysis with transition metal complexes: Applications in organic synthesis. Chem. Rev..

[13] Nicewicz D.A., Nguyen T.M. (2014). Recent applications of organic dyes as photoredox catalysts in organic synthesis. ACS Catal..

[14] Angnes R.A., Li Z., Correia C.R.D., Hammond G.B. (2015). Recent synthetic additions to the visible light photoredox catalysis toolbox. Org. Biomol. Chem..

[15] Shaw M.H., Twilton J., MacMillan D.W.C. (2016). Photoredox catalysis in organic chemistry. J. Org. Chem..

[16] Crisenza G.E.M., Melchiorre P. (2020). Chemistry glows green with photoredox catalysis. Nat. Commun..

[17] Mateus-Ruiz J.B., Cordero-Vargas A. (2020). Visible-light-mediated photoredox reactions in the total synthesis of natural products. Synthesis.

[18] Uygur M., Garcia Mancheño O. (2019). Visible light-mediated organophotocatalyzed C-H bond functionalization reactions. Org. Biomol. Chem..

[19] Revathi L., Ravindar L., Fang W.-Y., Rakesh K.P., Qin H.-L. (2018). Visible light-induced C-H bond functionalization: A critical review. Adv. Synth. Catal..

[20] Zhang Y., Schilling W., Das S. (2019). Metal-free photocatalysts for C-H bond oxygenation reactions with oxygen as the oxidant. ChemSusChem.

[21] Zhang X., Rakesh K.P., Ravindar L., Qin H.-L. (2018). Visible-light initiated aerobic oxidations: A critical review. Green Chem..

[22] Resende D.I.S.P., Durães F., Maia M., Sousa E., Pinto M.M.M. (2020). Recent advances in the synthesis of xanthones and azaxanthones. Org. Chem. Front..

[23] Ramakrishnan S., Paramewaran S., Nasir N.M. (2021). Synthetic approaches to biologically active xanthones: An update. Chem. Pap..

[24] Klein-Júnior L.C., Campos A., Niero R., Corrêa R., Vander Heyden Y., Cechinel Filho V. (2020). Xanthones and cancer: From natural sources to mechanisms of action. Chem. Biodiversity.

[25] Feng Z., Lu X., Gan L., Zhang Q., Lin L. (2020). Xanthones, a promising anti-inflammatory scaffold: Structure, activity, and drug likeness analysis. Molecules.

[26] Ng I.M.J., Chua C.L.L. (2019). The potential of xanthones as a therapeutic option in macrophage-associated inflammatory diseases. Pharmacogn. Rev..

[27] Bedi P., Gupta R., Pramanik T. (2018). Synthesis and biological properties of pharmaceutically important xanthones and benzoxanthone analogs: A brief review. Asian J. Pharm. Clin. Res..

[28] Paiva A.M., Pinto M.M., Sousa E. (2013). A century of thioxanthones: Through synthesis and biological applications. Curr. Med. Chem..

[29] Alwan W.S., Rane R.A., Amritkar A.A., Naphade S.S., Yerigiri M.C., Karpoormath R., Mahajan A.A. (2015). Acridone-based antitumor agents: A mini-review. Anti-Cancer Agents Med. Chem..

[30] Sepúlveda C.S., Fascio M.L., García C.C., D’Accorso N.B., Damonte E.B. (2013). Acridones as antiviral agents: Synthesis, chemical and biological properties. Curr. Med. Chem..

[31] Cholewiński G., Dzierzbicka K., Kołodziejczyk A.M. (2011). Natural and synthetic acridines/acridones as antitumor agents, their biological activities and methods of synthesis. Pharmacol. Rep..

[32] Vanover E., Huang Y., Xu L., Newcomb M., Zhang R. (2010). Photocatalytic aerobic oxidation by a bis-porphyrin-ruthenium(IV) μ-oxo dimer: Observation of a putative porphyrin-ruthenium(V)-oxo intermediate. Org. Lett..

[33] Gupta S.K., Choudhury J. (2017). A mixed *N*-heterocyclic carbene/2,2’-bipyridine-supported robust ruthenium(II) oxidation precatalyst for benzylic C-H oxidation. ChemCatChem.

[34] Shing K.-P., Cao B., Liu Y., Lee H.K., Li M.-D., Phillips D.L., Chang X.-Y., Che C.-M. (2018). Arylruthenium(III) porphyrin-catalyzed C-H oxidation and epoxidation at room temperature and [Ru^V^(Por)(O)(Ph)] intermediate by spectroscopic analysis and density functional theory calculations. J. Am. Chem. Soc..

[35] Mühldorf B., Wolf R. (2016). C-H Photooxygenation of alkyl benzenes catalyzed by riboflavin tetraacetate and a non-heme iron catalyst. Angew. Chem. Int. Ed..

[36] Liu X., Lin L., Ye X., Tan C.-H., Jiang Z. (2017). Aerobic oxidation of benzylic sp^3^ C-H bonds through cooperative visible-light photoredox catalysis of *N*-hydroxyimide and dicyanopyrazine. Asian J. Org. Chem..

[37] Li S., Zhu B., Lee R., Qiao B., Jiang Z. (2018). Visible light-induced selective aerobic oxidative transposition of vinyl halides using a tetrahalogenoferrate(III) complex catalyst. Org. Chem. Front..

[38] Kumari S., Yadav D., Sharma S.K. (2020). Cu(II) Schiff base complex grafted guar gum: Catalyst for benzophenone derivatives synthesis. Appl. Catal. A.

[39] Finney L.C., Mitchell L.J., Moody C.J. (2018). Visible light mediated oxidation of benzylic sp^3^ C-H bonds using catalytic 1,4-hydroquinone, or its biorenewable glucoside, arbutin, as a pre-oxidant. Green Chem..

[40] Sarma D., Majumdar B., Sarma T.K. (2019). Visible-light induced enhancement in the multi-catalytic activity of sulfated carbon dots for aerobic carbon-carbon bond formation. Green Chem..

[41] Geng P., Tang Y., Pan G., Wang W., Hu J., Cai Y. (2019). A g-C_3_N_4_-based heterogeneous photocatalyst for visible light mediated aerobic benzylic C-H oxygenations. Green Chem..

[42] Pan D., Wang Y., Li M., Hu X., Sun N., Jin L., Hu B., Shen Z. (2019). Visible-light-induced aerobic oxidation of benzylic C(sp^3^)-H of alkylarenes promoted by DDQ, *tert*-butyl nitrite, and acetic acid. Synlett.

[43] Xiang M., Xin Z.-K., Chen B., Tung C.-H., Wu L.-Z. (2017). Exploring the reducing ability of organic Dye (Acr^+^-Mes) for fluorination and oxidation of benzylic C(sp^3^)-H bonds under visible light irradiation. Org. Lett..

[44] Tolba A.H., Vávra F., Chudoba J., Cibulka R. (2020). Tuning flavin-based photocatalytic systems for application in the mild chemoselective aerobic oxidation of benzylic substrates. Eur. J. Org. Chem..

[45] Jung J., Ohkubo K., Prokop-Prigge K.A., Neu H.M., Goldberg D.P., Fukuzumi S. (2013). Photochemical oxidation of a manganese(III) complex with oxygen and toluene derivatives to form a manganese(V)-oxo complex. Inorg. Chem..

[46] Pandey G., Jadhav D., Tiwari S.K., Singh B. (2014). Visible light photoredox catalysis: Investigation of distal sp^3^ C-H functionalization of tertiary amines for alkylation reaction. Adv. Synth. Catal..

[47] Fukuzumi S., Ohkubo K. (2014). Organic synthetic transformations using organic dyes as photoredox catalysts. Org. Biomol. Chem..

[48] Ravelli D., Fagnoni M. (2012). Dyes as visible light photoredox organocatalysts. ChemCatChem.

[49] Li P.-C., Wang T.-S., Lee G.-H., Liu Y.-H., Wang Y., Chen C.-T., Chao I. (2002). Theoretical study and X-ray determination of bianthrones: Long C-C bond length and preferred gauche conformation. J. Org. Chem..

[50] Silva E., Edwards A.M. (2006). Flavins: Photochemistry and Photobiology.

[51] König B., Kömmel S., Cibulka R., König B. (2013). Flavin Photocatalysis. Chemical Photocatalysis.

[52] de Gonzalo G., Fraaije M.W. (2013). Recent developments in flavin-based catalysis. ChemCatChem.

[53] König B., Kömmel S., Svodobová E., Cibulka R. (2018). Flavin photocatalysis. Phys. Sci. Rev..

[54] Metternich J.B., Mudd R.J., Gilmour R., König B. (2019). Flavins in Photocatalysis. Science of Synthesis: Photocatalysis in Organic Synthesis.

[55] Teegardin K., Day J.I., Chan J., Weaver J. (2016). Advances in photocatalysis: A microreview of visible light mediated ruthenium and iridium catalyzed organic transformations. Org. Process. Res. Dev..

[56] Angerani S., Winssinger N. (2019). Visible light photoredox catalysis using ruthenium complexes in chemical biology. Chem. Eur. J..

[57] Yi H., Bian C., Hu X., Niu L., Lei A. (2015). Visible light mediated efficient oxidative benzylic sp^3^ C-H to ketone derivatives obtained under mild conditions using O_2_. Chem. Commun..

[58] Sikorska E., Sikorski M., Steer R.P., Wilkinson F., Worrall D.R. (1998). Efficiency of singlet oxygen generation by alloxazines and isoalloxazines. J. Chem. Soc. Faraday Trans..

[59] Sahoo M.K., Jaiswal G., Rana J., Balaraman E. (2017). Organo-photoredox catalyzed oxidative dehydrogenation of *N*-heterocycles. Chem. Eur. J..

[60] Yu K., Zhang H., Su C., Zhu Y. (2020). Visible-light-promoted efficient aerobic dehydrogenation of *N*-heterocycles by a tiny organic semiconductor under ambient conditions. Eur. J. Org. Chem..

[61] Larson R.A., Stackhouse P.L., Crowley T.O. (1992). Riboflavin tetraacetate: A potentially useful photosensitizing agent for the treatment of contaminated waters. Environ. Sci. Technol..

[62] Böβ E., Hillringhaus T., Nitsch J., Klussmann M. (2011). Lewis acid-catalyzed one pot synthesis of substituted xanthenes. Org. Biomol. Chem..

[63] Yuan Y., Qiao J., Cao Y., Tang J., Wang M., Ke G., Lu Y., Liu X., Lei A. (2019). Exogenous-oxidant-free electrochemical oxidative C-H phosphonylation with hydrogen evolution. Chem. Commun..

[64] Pintér A., Sud A., Sureshkumar D., Klussmann M. (2010). Autoxidative carbon-carbon bond formation from carbon-hydrogen bonds. Angew. Chem. Int. Ed..

[65] Hernandez-Olmos V., Abdelrahman A., El-Tayeb A., Freudendahl D., Weinhausen S., Müller C.E. (2012). *N*-Substituted phenoxazine and acridone derivatives: Structure-activity relationships of potent P2X4 receptor antagonists. J. Med. Chem..

[66] Iwama Y., Noro T., Okano K., Cho H., Tokuyama H. (2014). Formation of xanthone oxime and related compounds using a combination of *tert*-butyl nitrite and potassium hexamethyldisilazide. Heterocycles.

[67] Mulder P., Litwinienko G., Lin S., MacLean P.D., Barclay L.R.C., Ingold K.U. (2006). The L-type calcium channel blockers, Hantzsch 1,4-dihydropyridines, are not peroxyl radical-trapping, chain-breaking antioxidants. Chem. Res. Toxicol..

[68] Zhao J., Larock R.C. (2007). Synthesis of xanthones, thioxanthones, and acridones by the coupling of arynes and substituted benzoates. J. Org. Chem..

[69] Zhao J., Yue D., Campo M.A., Larock R.C. (2007). An aryl to imidoyl palladium migration process involving intramolecular C-H activation. J. Am. Chem. Soc..

[70] Xiong Z., Zhang X., Li Y., Peng X., Fu J., Guo J., Xie F., Jiang C., Lin B., Liu Y. (2018). Syntheses of 12*H*-benzo[*a*]xanthen-12-ones and benzo[*a*]acridin-12(7*H*)-ones through Au(I)-catalyzed Michael addition/6-*endo*-trig cyclization/aromatization cascade annulation. Org. Biomol. Chem..

[71] Janke J., Villinger A., Ehlers P., Langer P. (2019). Synthesis of acridones by palladium-catalyzed Buchwald-Hartwig amination. Synlett.

[72] Bi X., Tang T., Meng X., Gou M., Liu X., Zhao P. (2020). Aerobic oxidative dehydrogenation of *N*-heterocycles over OMS-2-based nanocomposite catalysts: Preparation, characterization and kinetic study. Catal. Sci. Technol..

