# Supplementary material for: Synthesis of Xanthones, Thioxanthones and Acridones by a Metal-Free Photocatalytic Oxidation Using Visible Light and Molecular Oxygen
Source: Molecules. 2021 Feb 12;26(4):974. doi: 10.3390/molecules26040974 (PMC7918112; doi:10.3390/molecules26040974)
Supplement: Supplementary file 1 [file molecules-26-00974-s001.pdf]

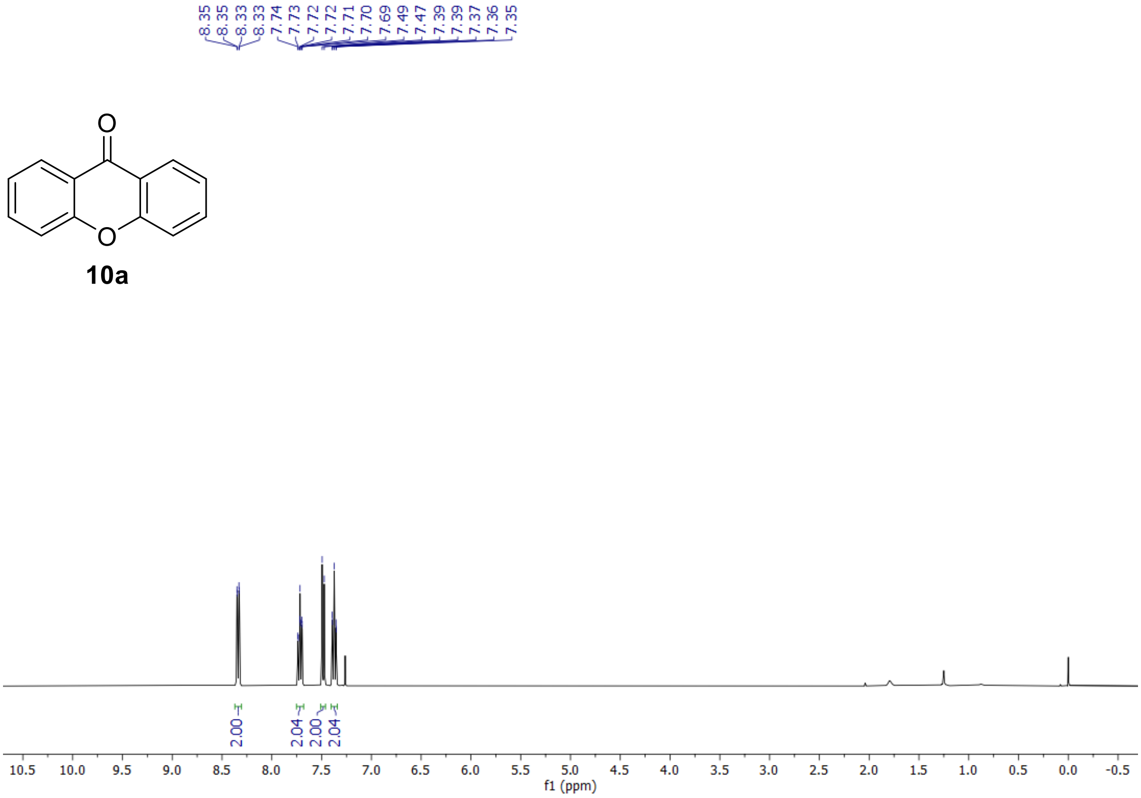

Figure S1. ^{1}H NMR spectrum ($CDCl_3$, 400 MHz) of compound **10a**.

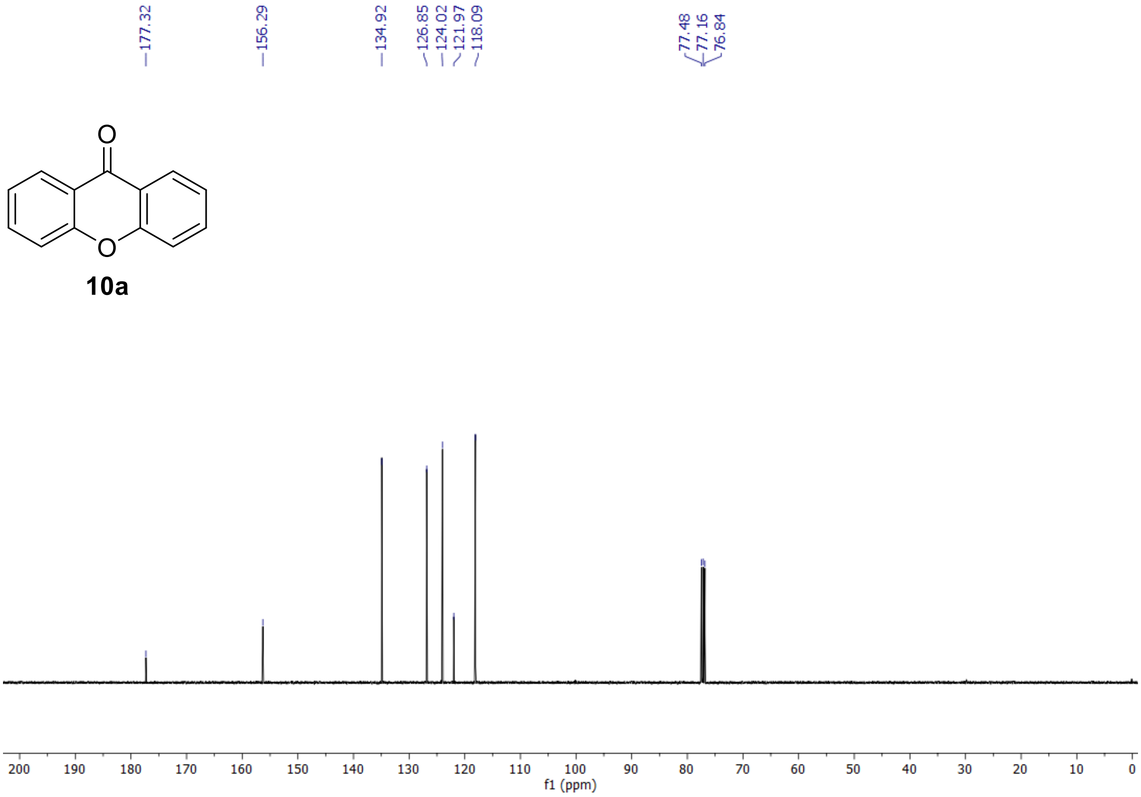

Figure S2. ^{13}C NMR spectrum ($CDCl_3$, 101 MHz) of compound **10a**.

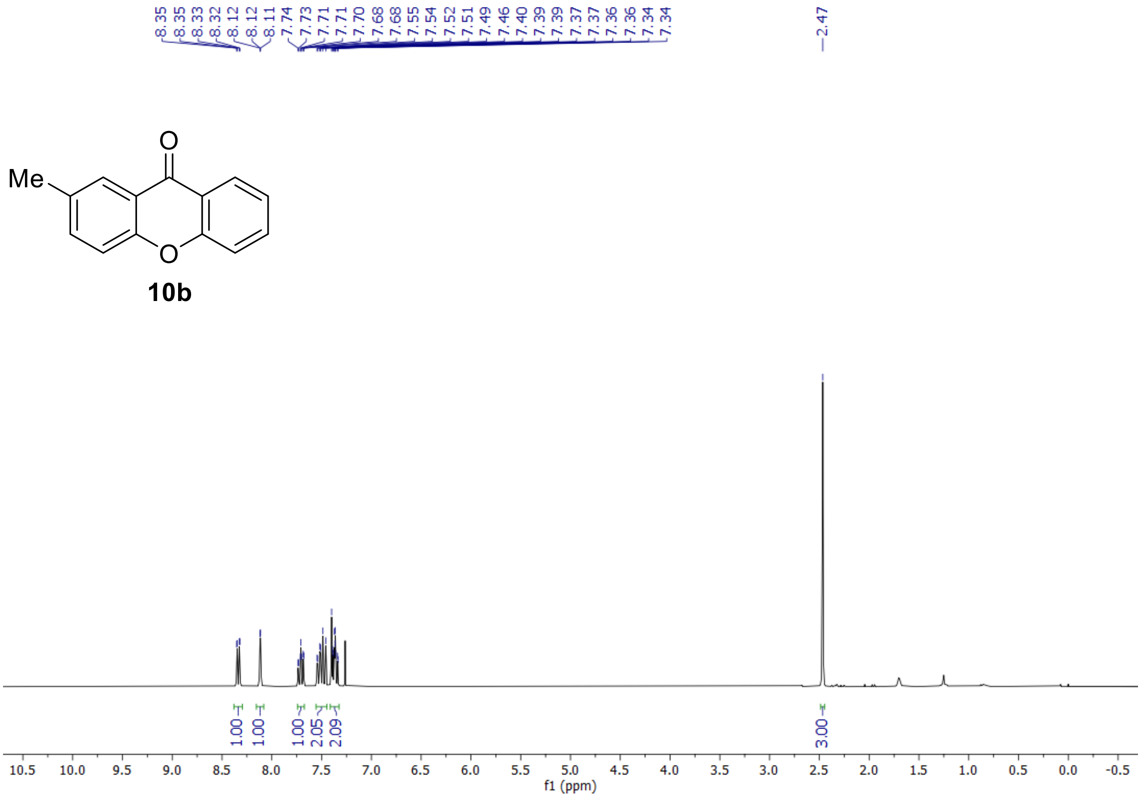

Figure S3. ^{1}H NMR spectrum ($CDCl_3$, 300 MHz) of compound **10b**.

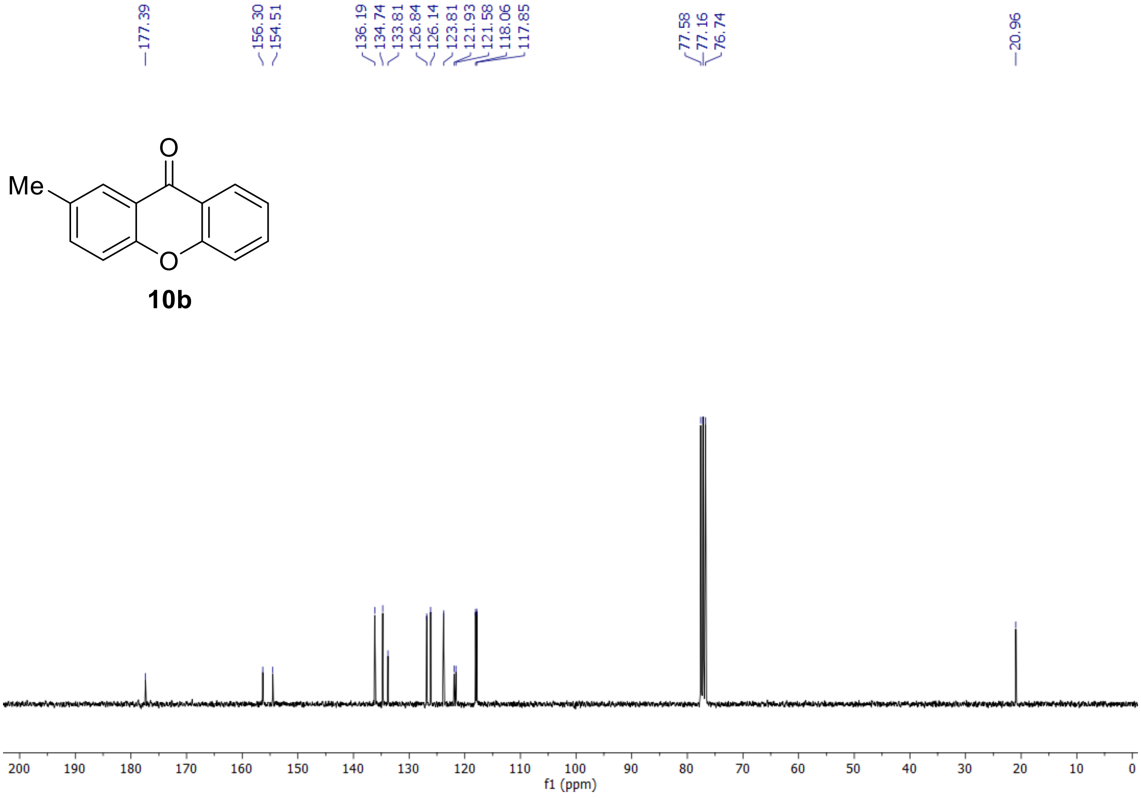

Figure S4. ^{13}C NMR spectrum ($CDCl_3$, 101 MHz) of compound **10b**.

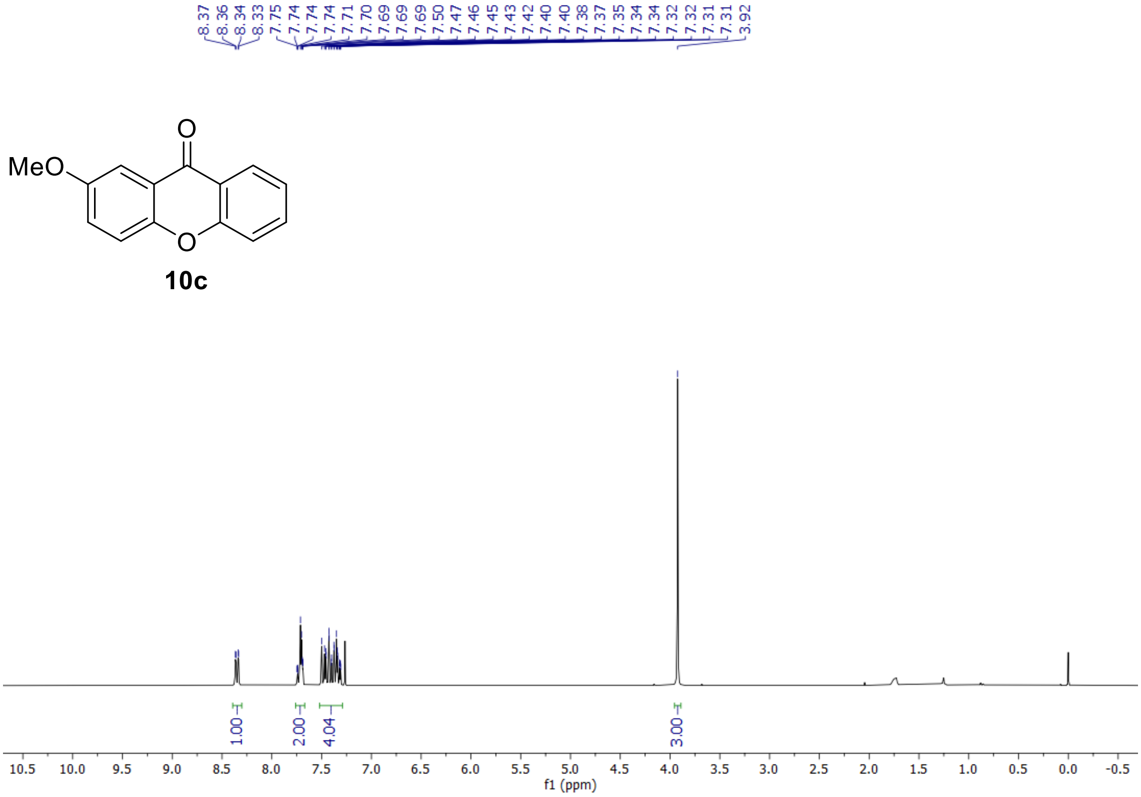

Figure S5. ^{1}H NMR spectrum ($CDCl_3$, 300 MHz) of compound **10c**.

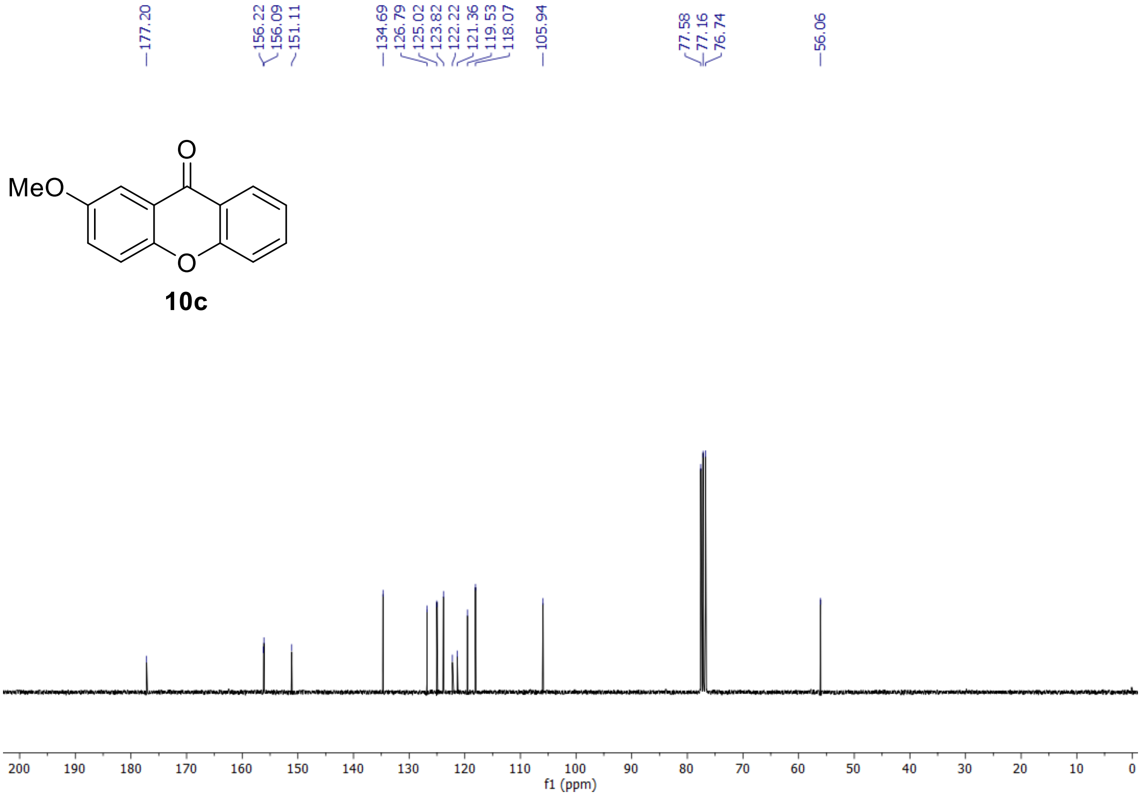

Figure S6. ^{13}C NMR spectrum ($CDCl_3$, 75 MHz) of compound **10c**.

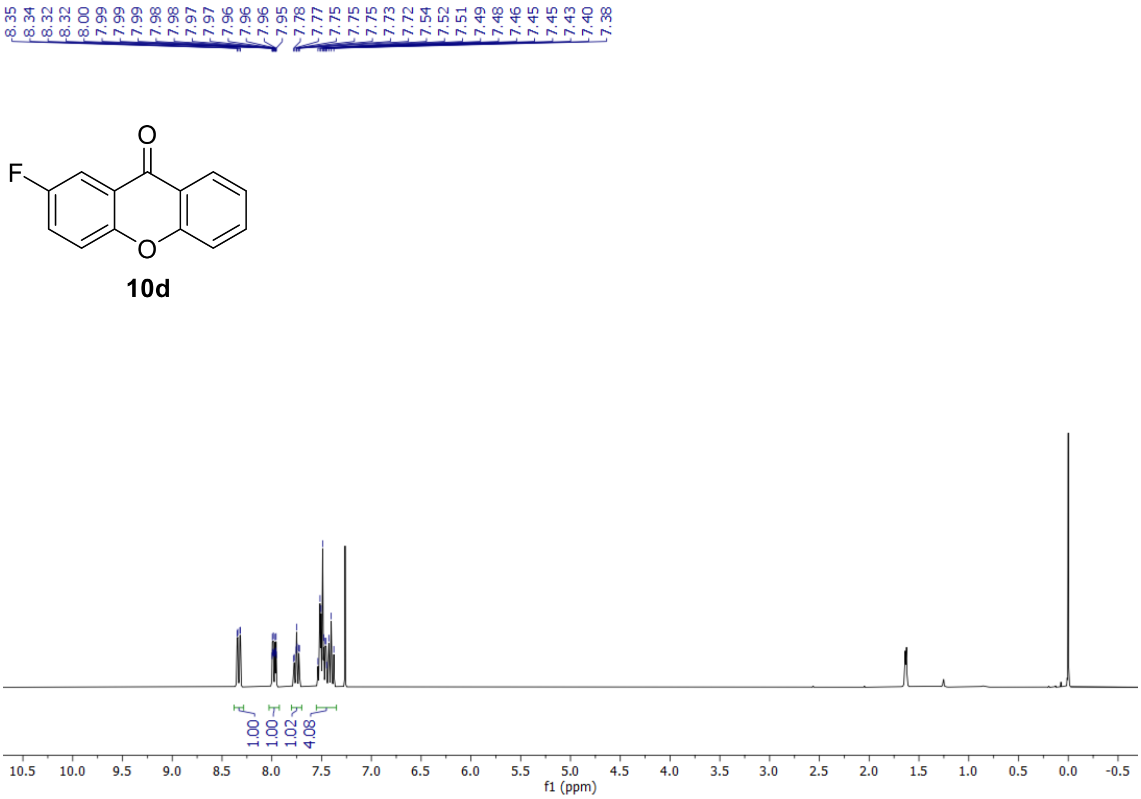

Figure S7. ^{1}H NMR spectrum ($CDCl_3$, 300 MHz) of compound **10d**.

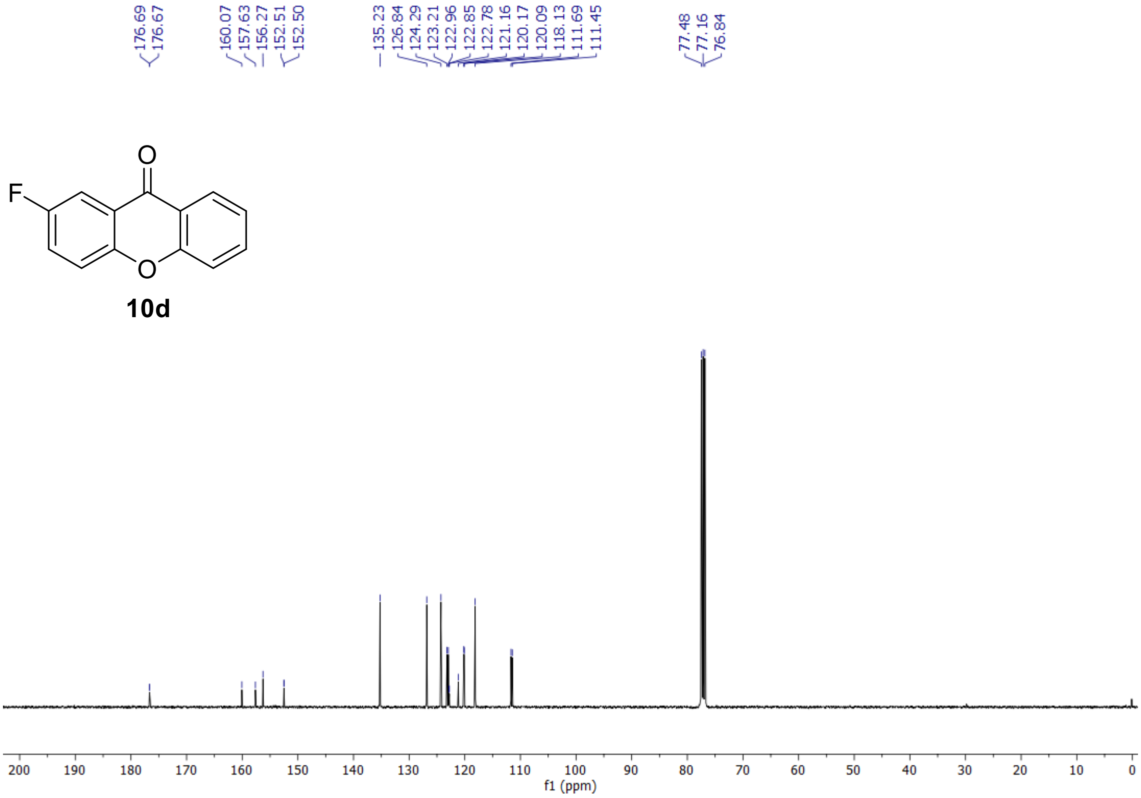

Figure S8. ^{13}C NMR spectrum ($CDCl_3$, 101 MHz) of compound **10d**.

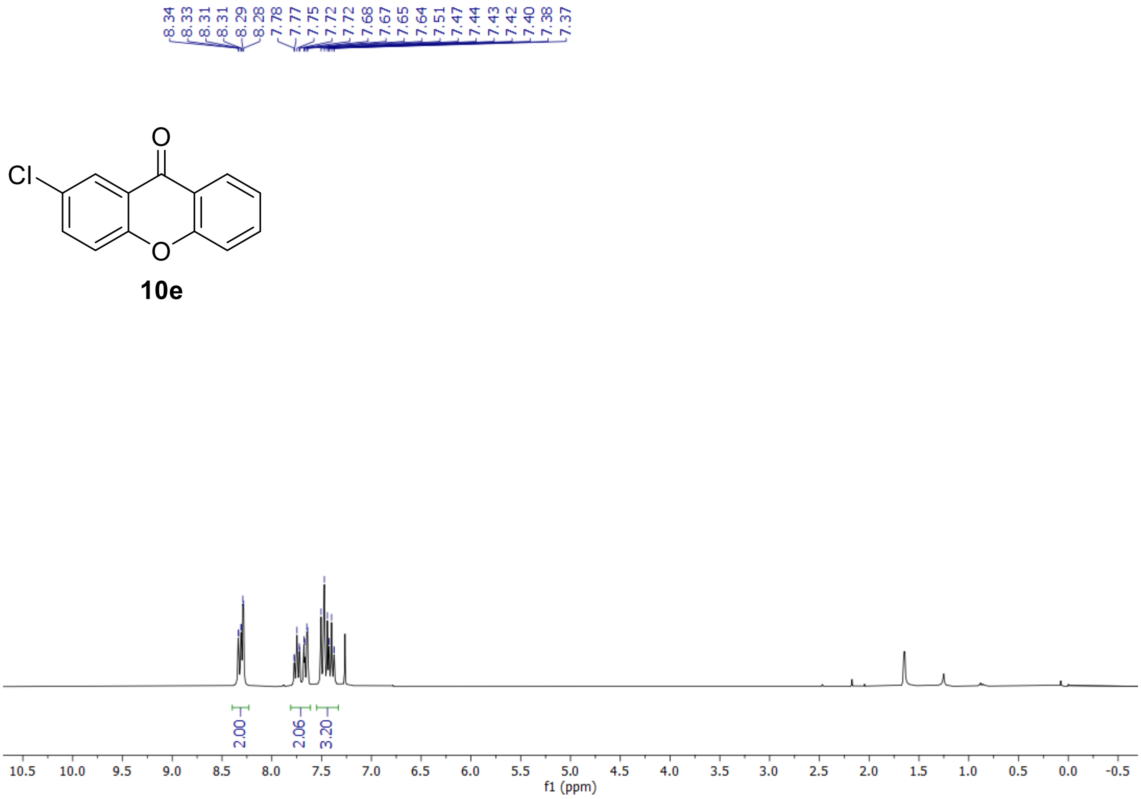

Figure S9. ^{1}H NMR spectrum ($CDCl_3$, 300 MHz) of compound **10e**.

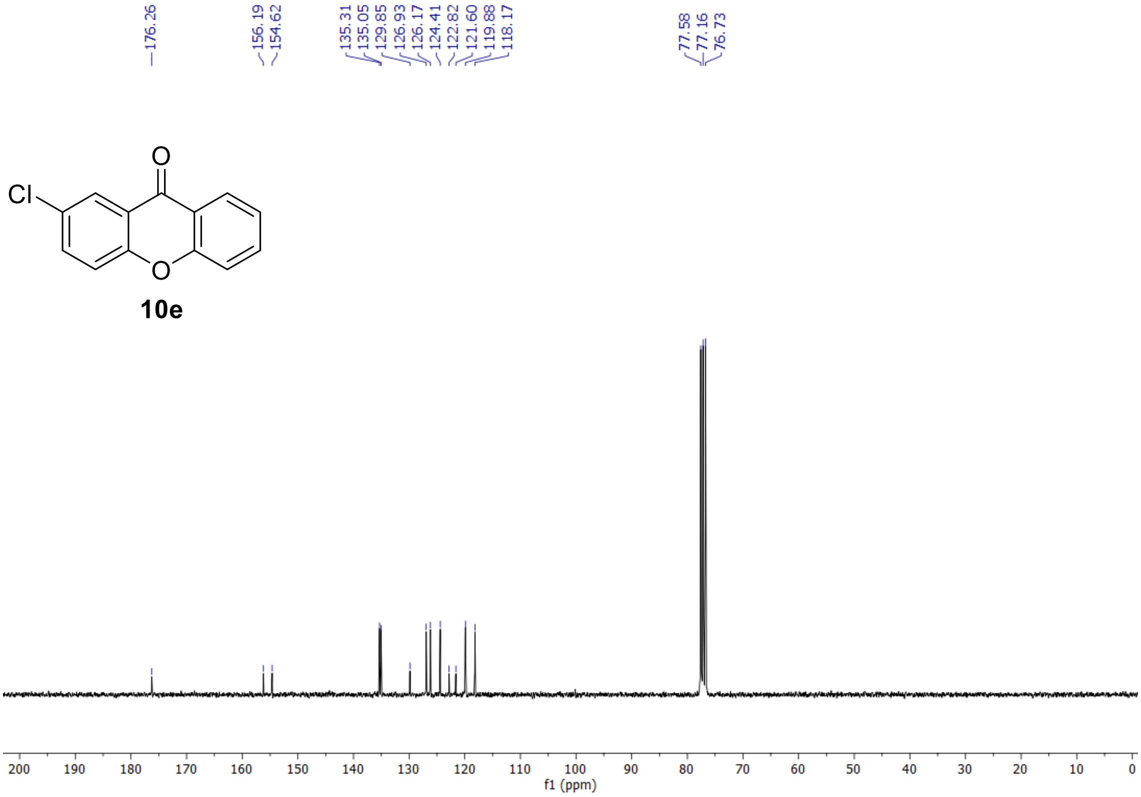

Figure S10. ^{13}C NMR spectrum ($CDCl_3$, 75 MHz) of compound **10e**.

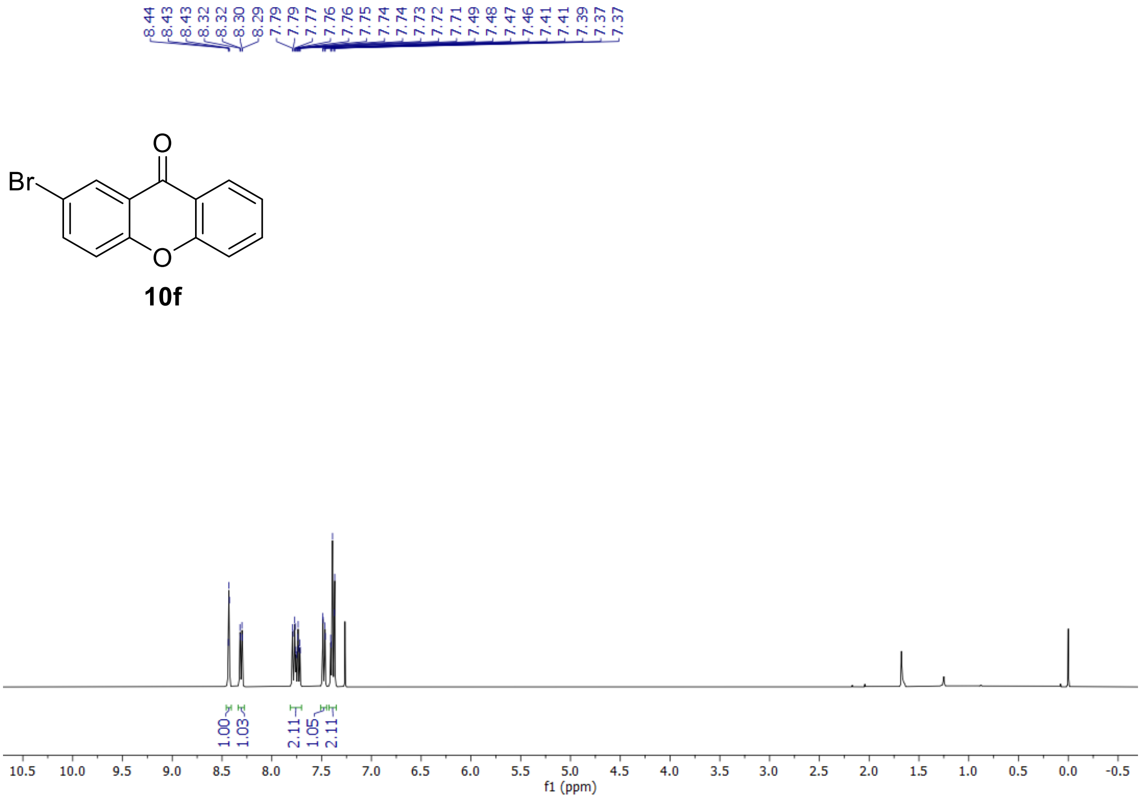

Figure S11. ^{1}H NMR spectrum ($CDCl_3$, 400 MHz) of compound **10f**.

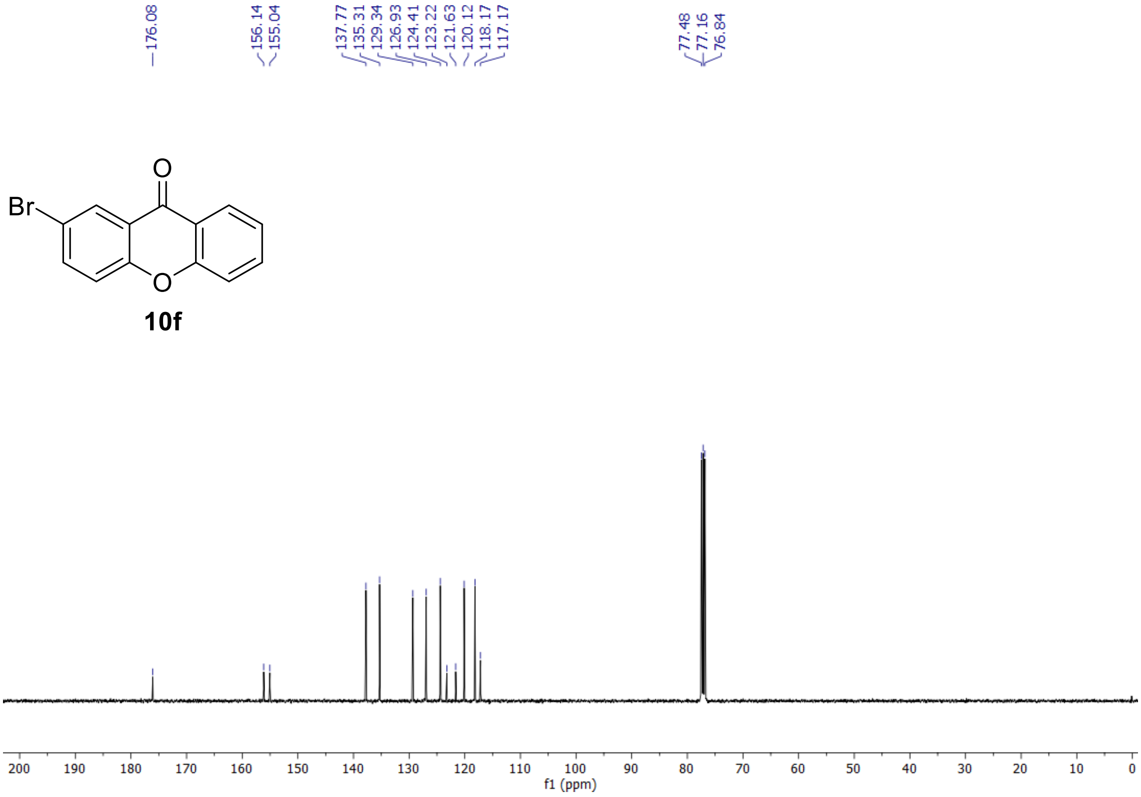

Figure S12. ^{13}C NMR spectrum ($CDCl_3$, 101 MHz) of compound **10f**.

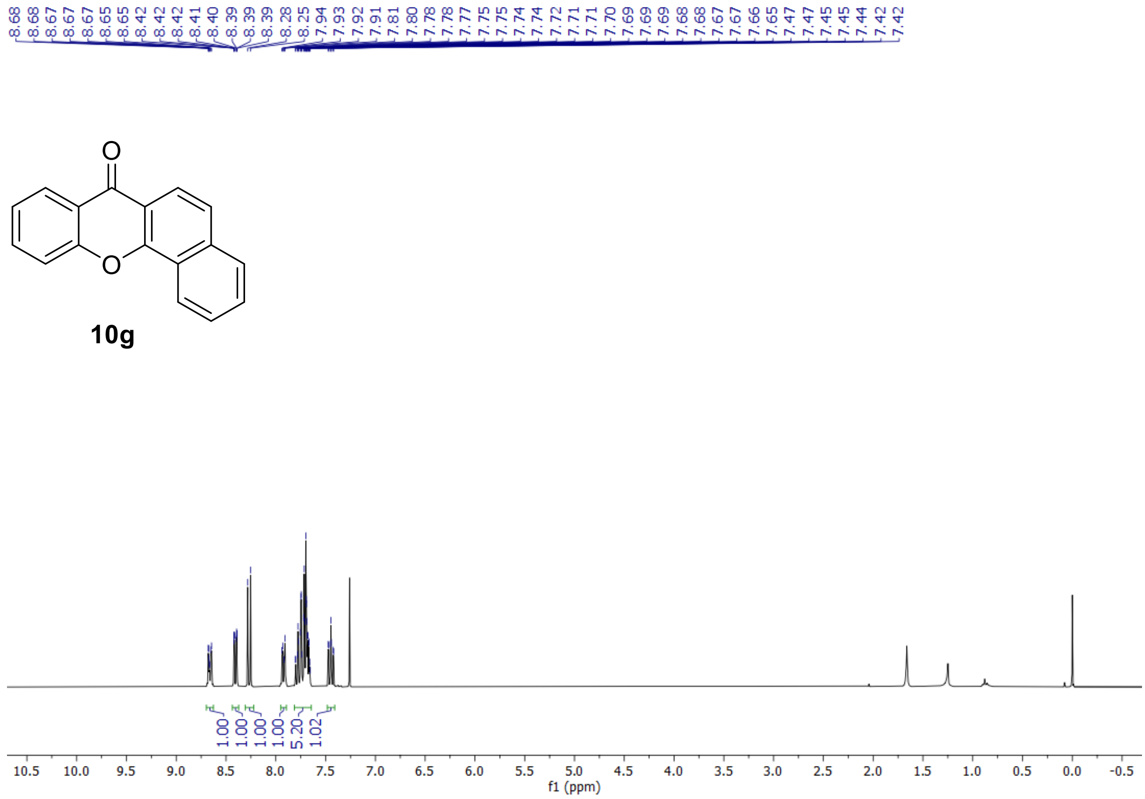

Figure S13. ^{1}H NMR spectrum ($CDCl_3$, 300 MHz) of compound **10g**.

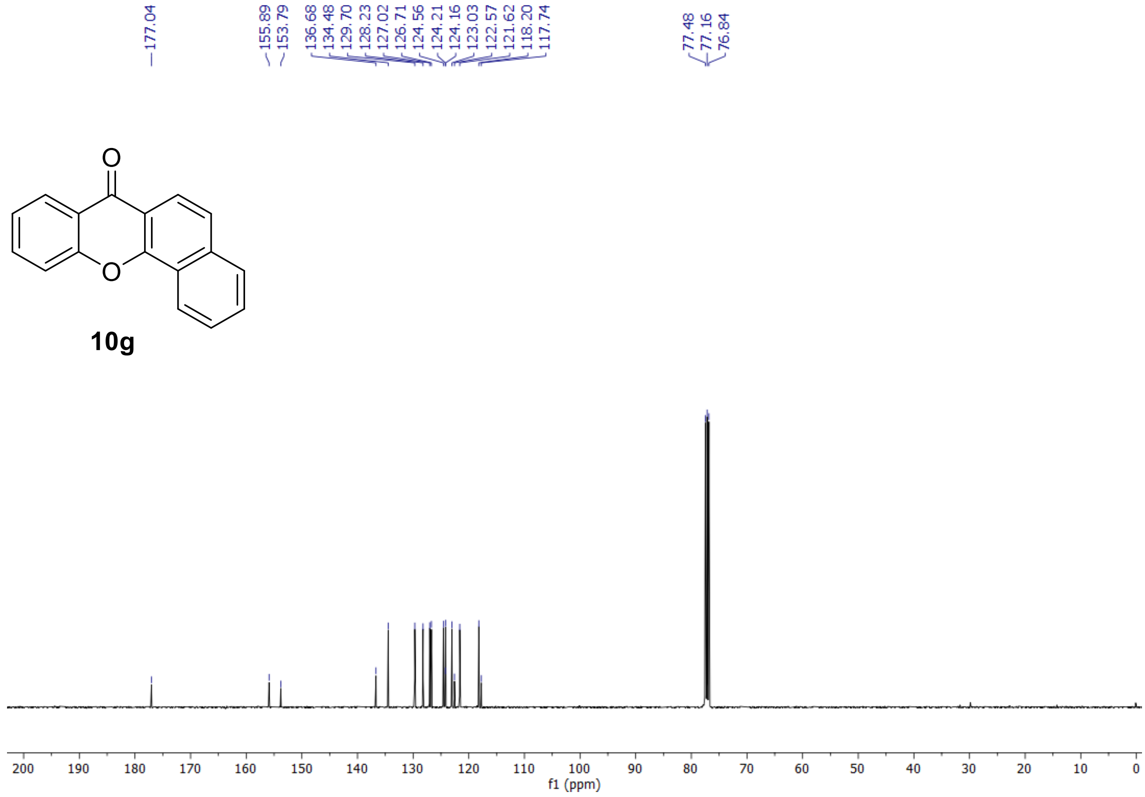

Figure S14. ^{13}C NMR spectrum ($CDCl_3$, 101 MHz) of compound **10g**.

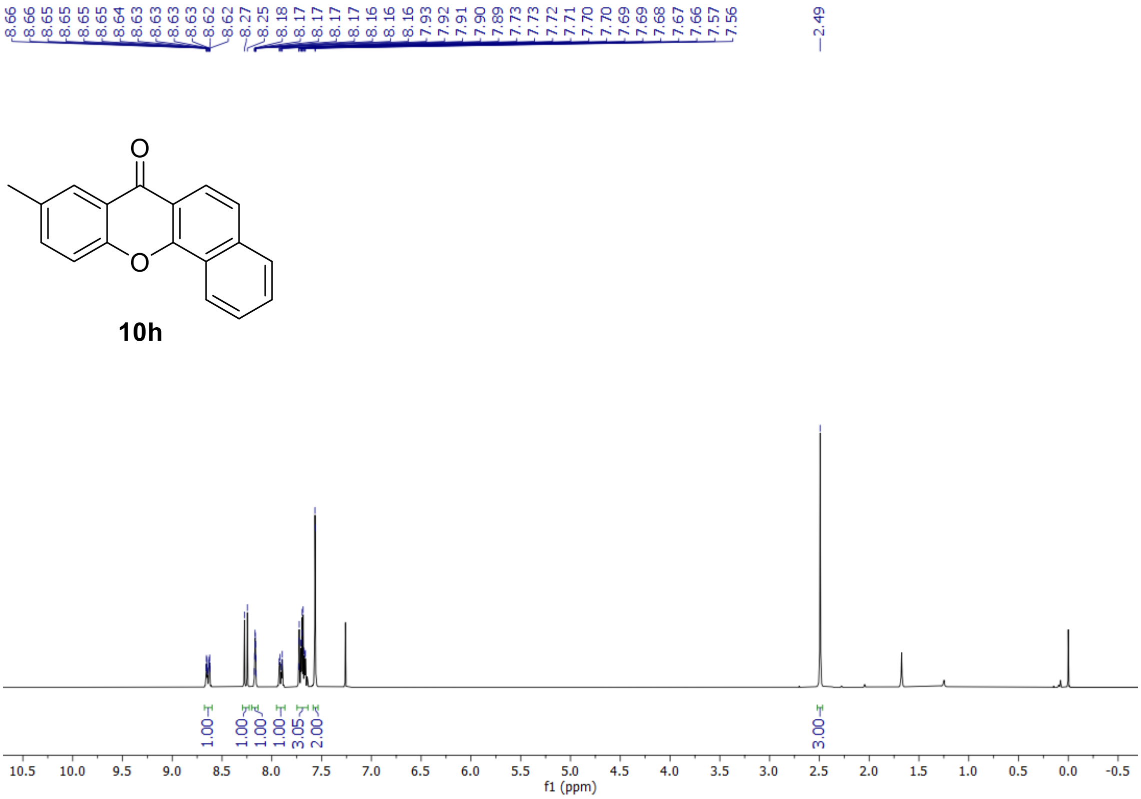

Figure S15. ^{1}H NMR spectrum ($CDCl_3$, 300 MHz) of compound **10h**.

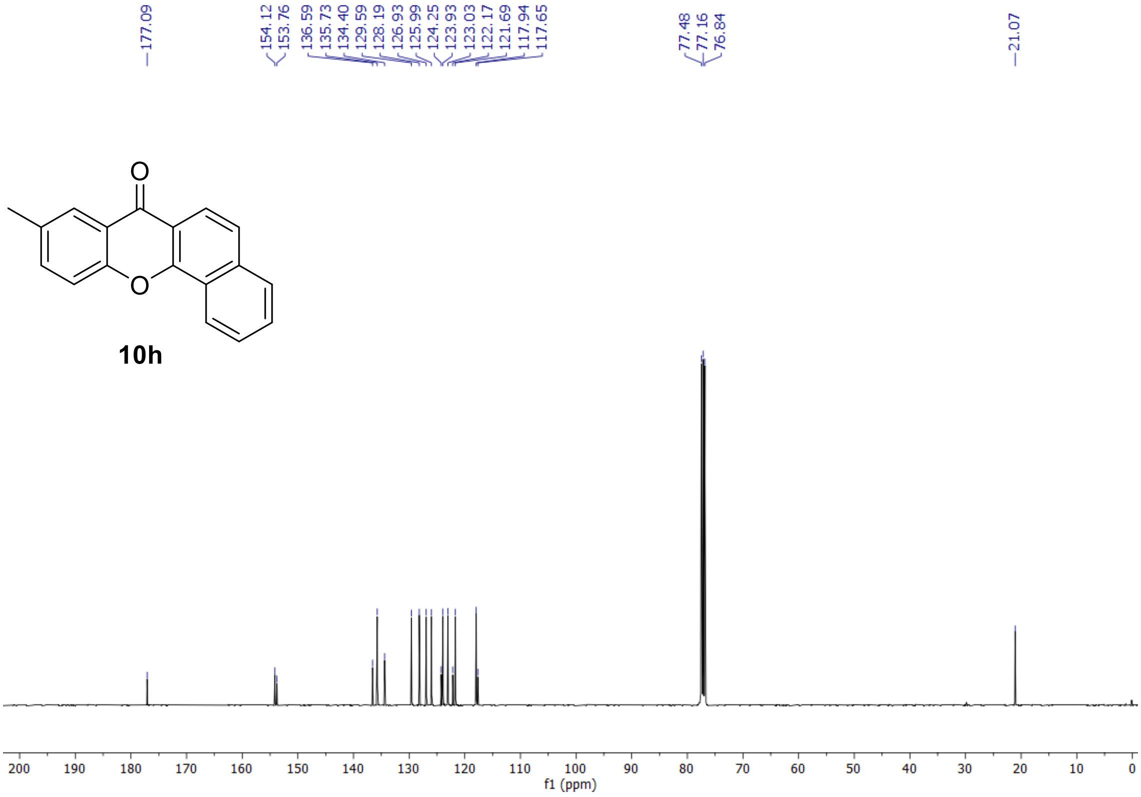

Figure S16. ^{13}C NMR spectrum ($CDCl_3$, 101 MHz) of compound **10h**.

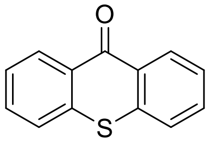

10i

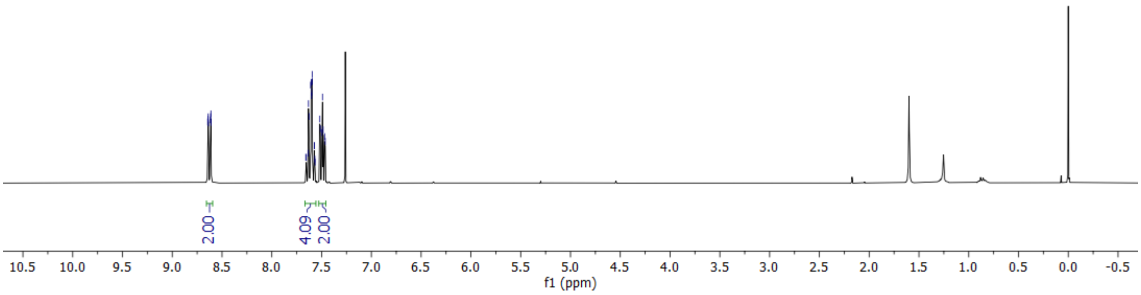

Figure S17. ^{1}H NMR spectrum ($CDCl_3$, 300 MHz) of compound **10i**.

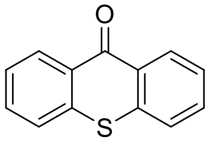

10i

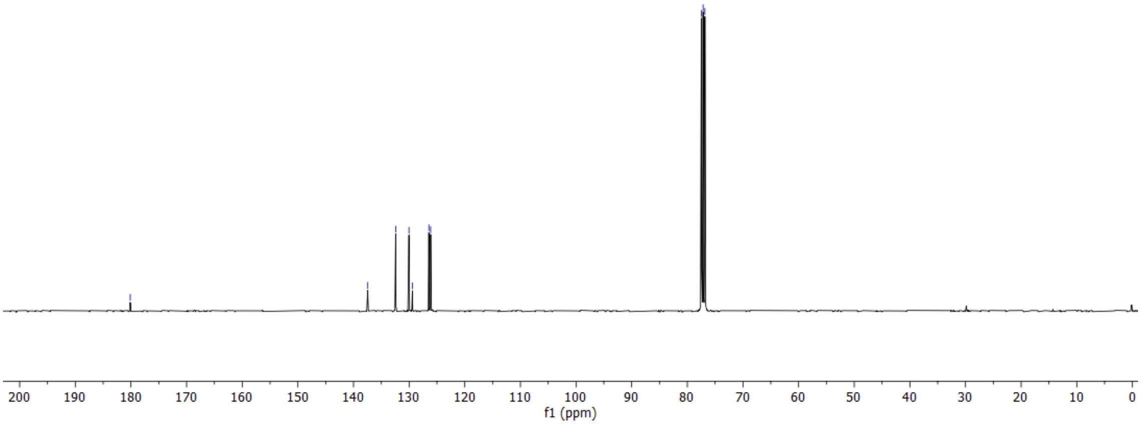

Figure S18. ^{13}C NMR spectrum ($CDCl_3$, 101 MHz) of compound **10i**.

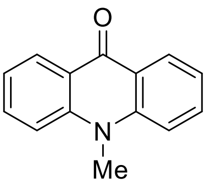

10j

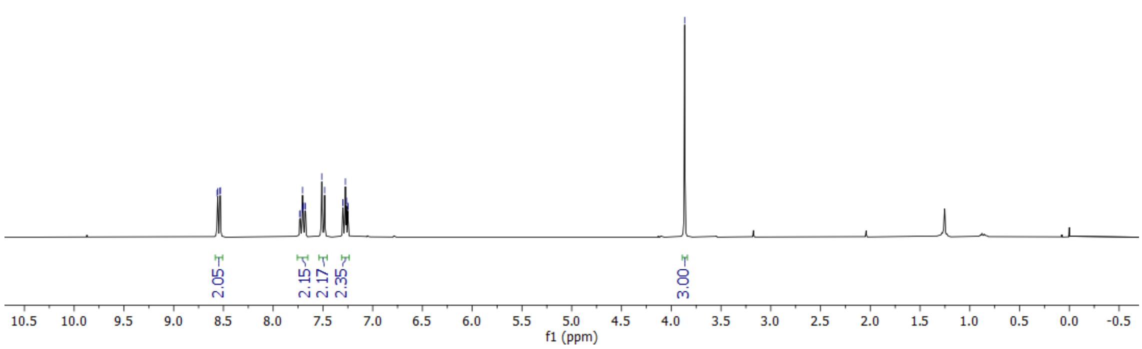

Figure S19. ^{1}H NMR spectrum ($CDCl_3$, 300 MHz) of compound **10j**.

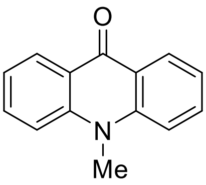

10j

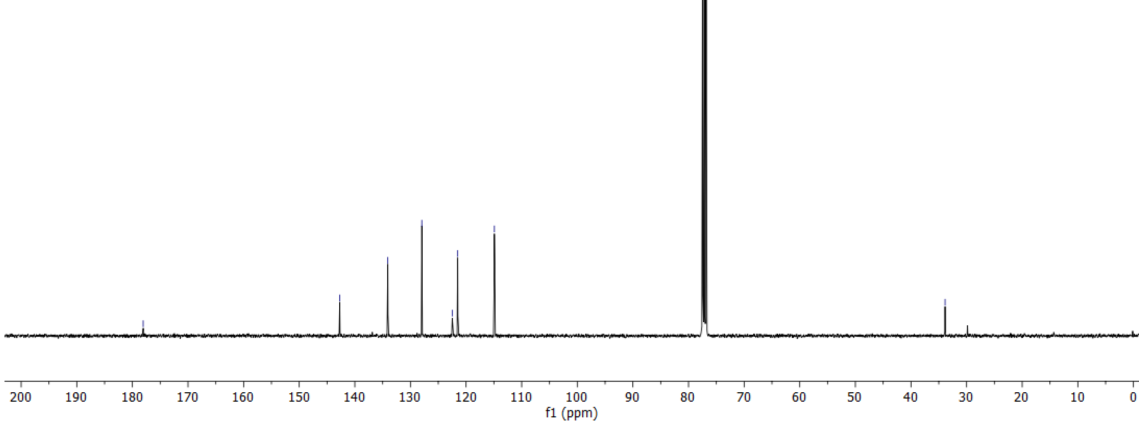

Figure S20. ^{13}C NMR spectrum ($CDCl_3$, 101 MHz) of compound **10j**.

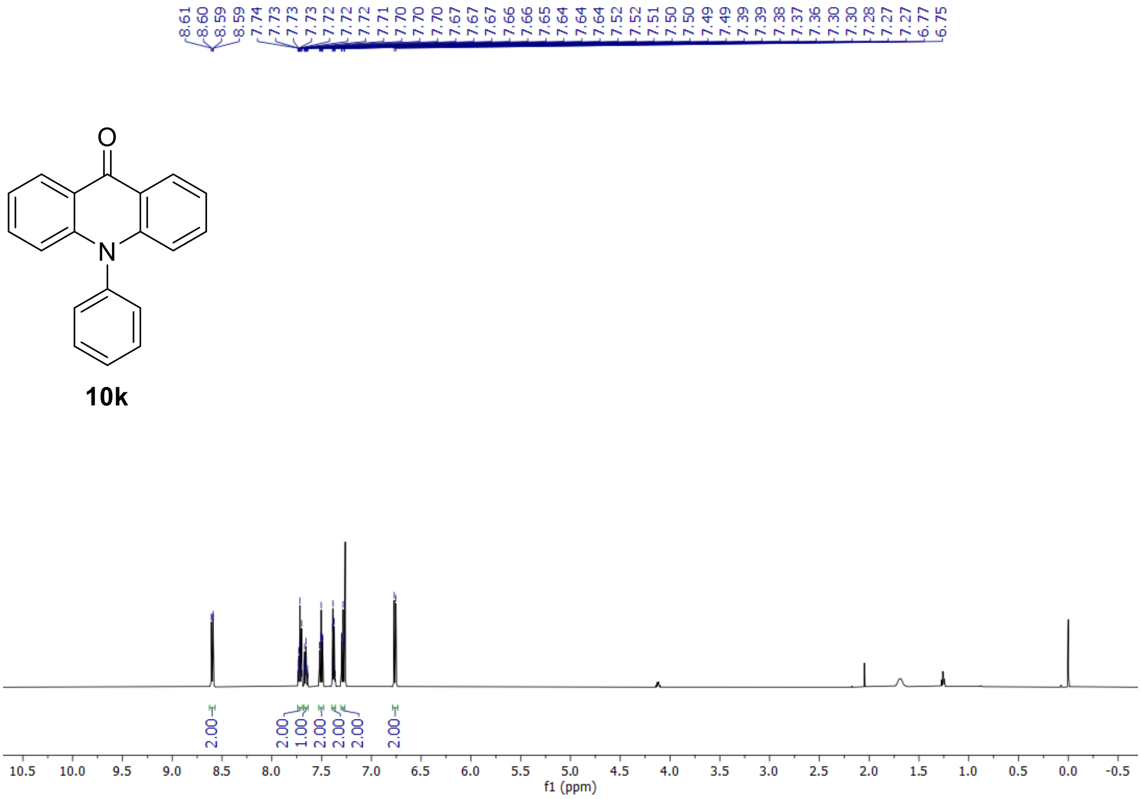

Figure S21. ^{1}H NMR spectrum ($CDCl_3$, 500 MHz) of compound **10k**.

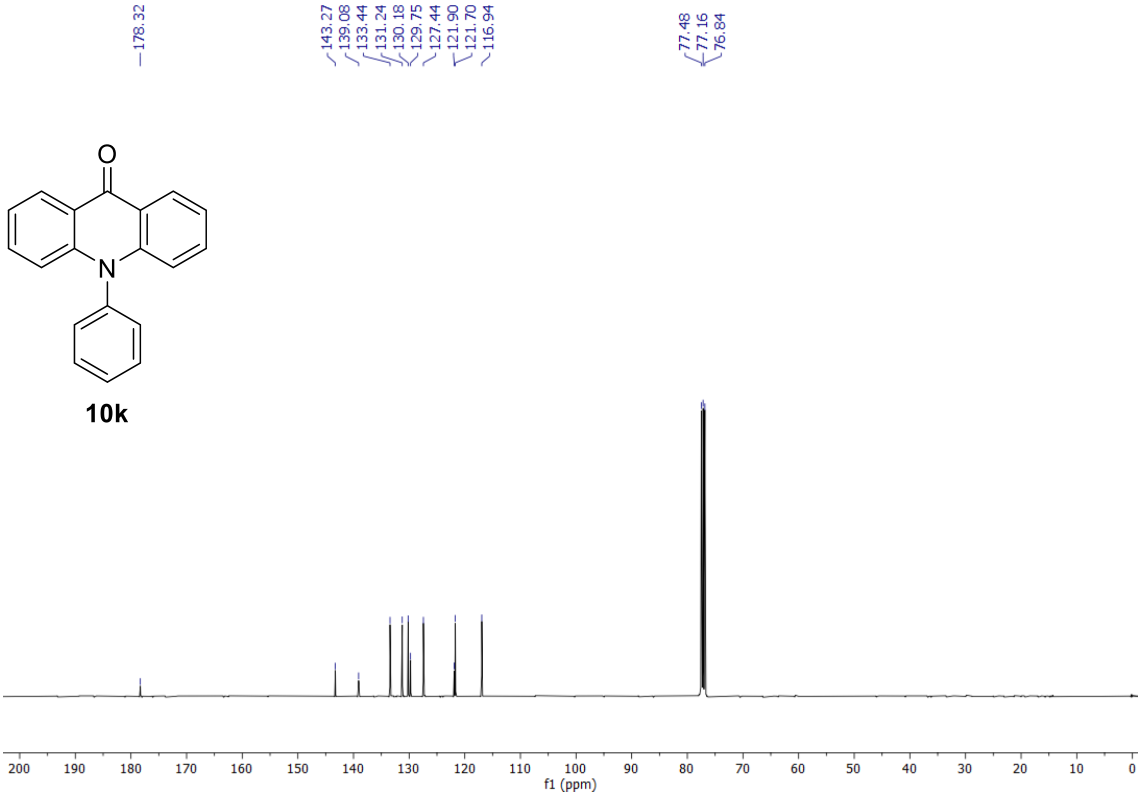

Figure S22. ^{13}C NMR spectrum ($CDCl_3$, 125 MHz) of compound **10k**.

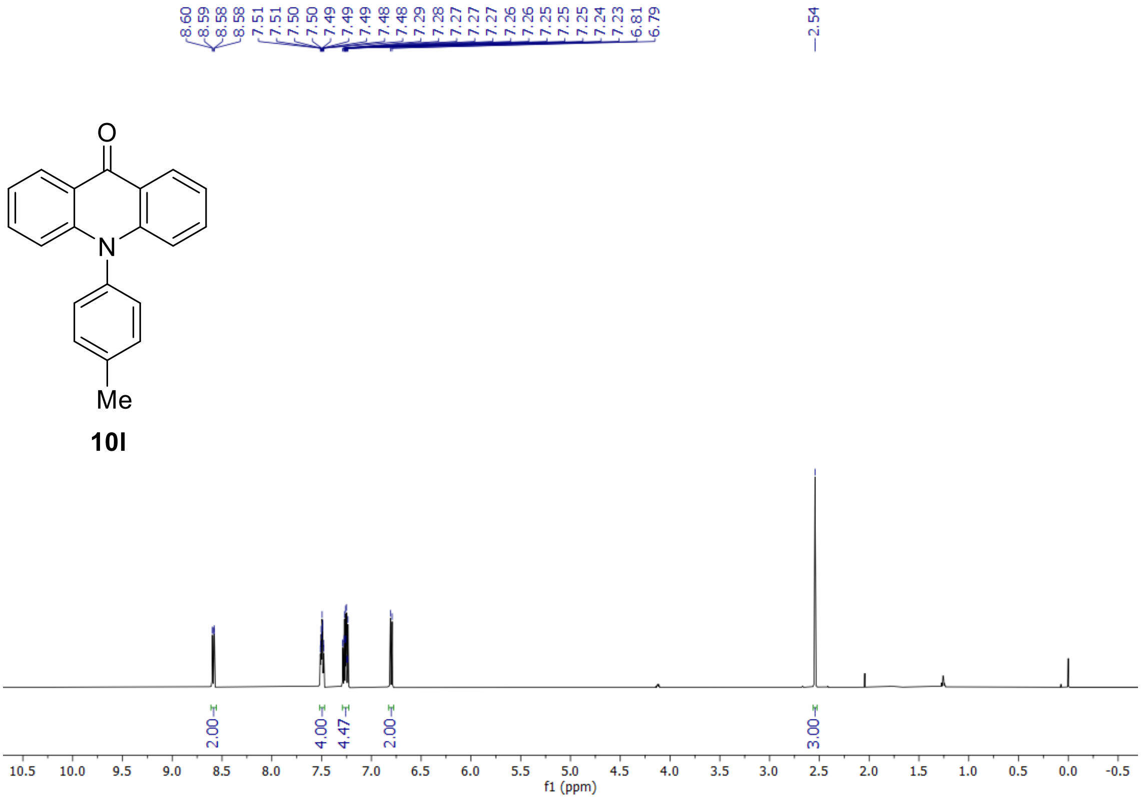

Figure S23. ^{1}H NMR spectrum ($CDCl_3$, 500 MHz) of compound **10l**.

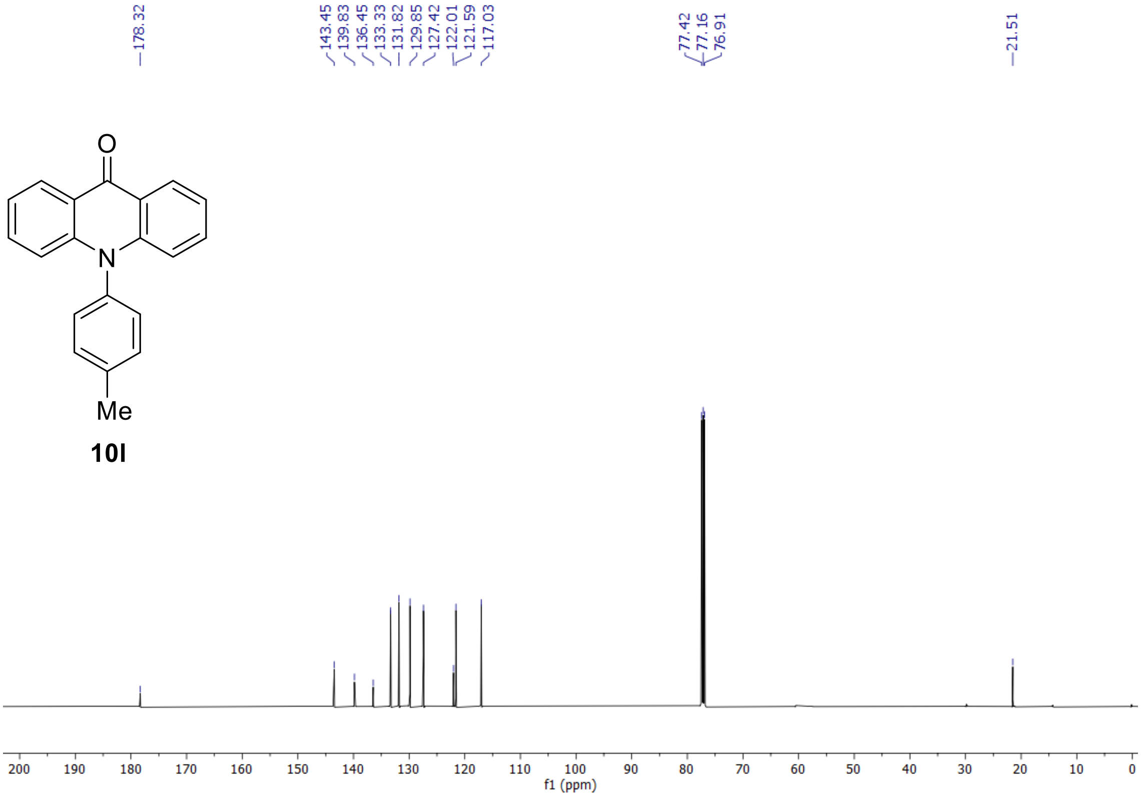

Figure S24. ^{13}C NMR spectrum ($CDCl_3$, 125 MHz) of compound **10l**.

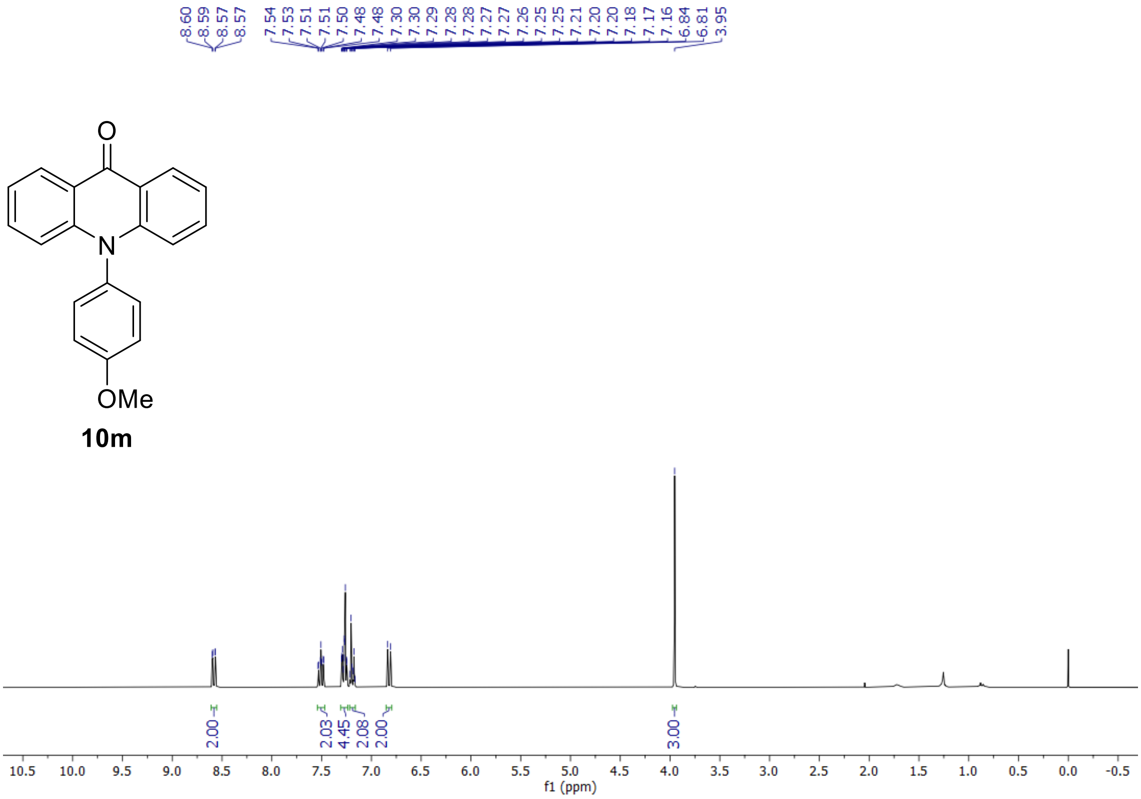

Figure S25. ^{1}H NMR spectrum ($CDCl_3$, 300 MHz) of compound **10m**.

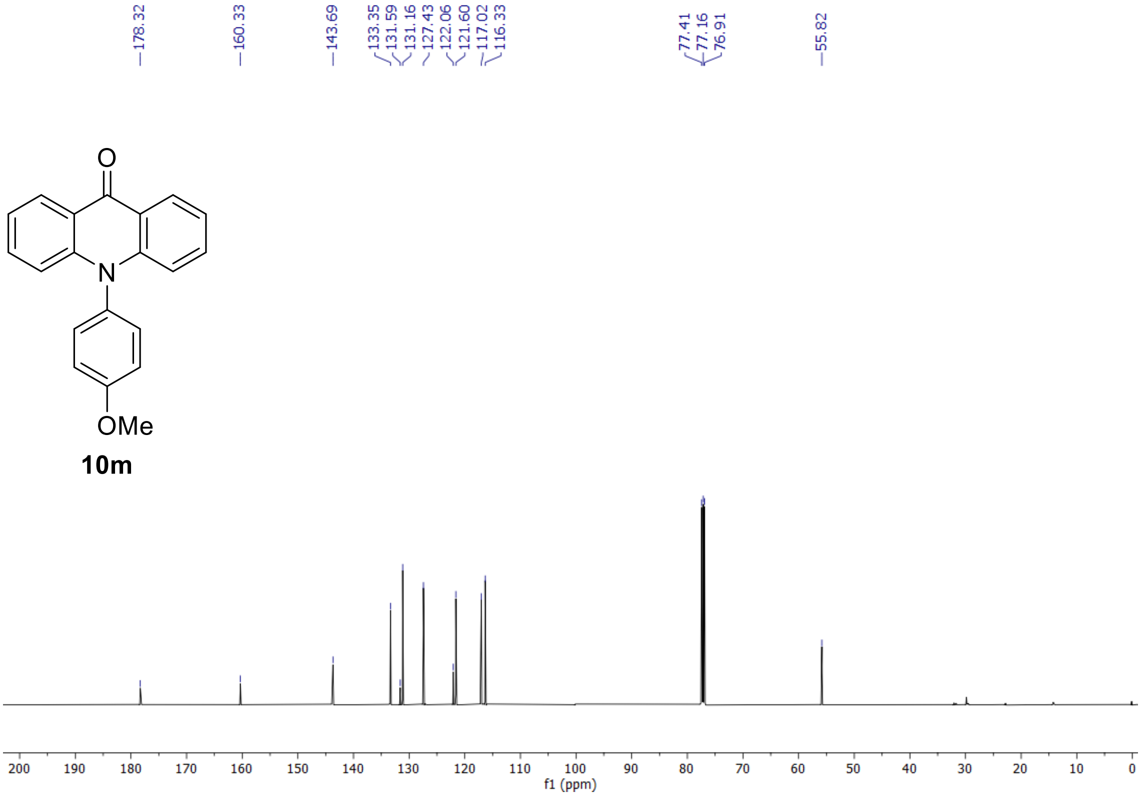

Figure S26. ^{13}C NMR spectrum ($CDCl_3$, 125 MHz) of compound **10m**.

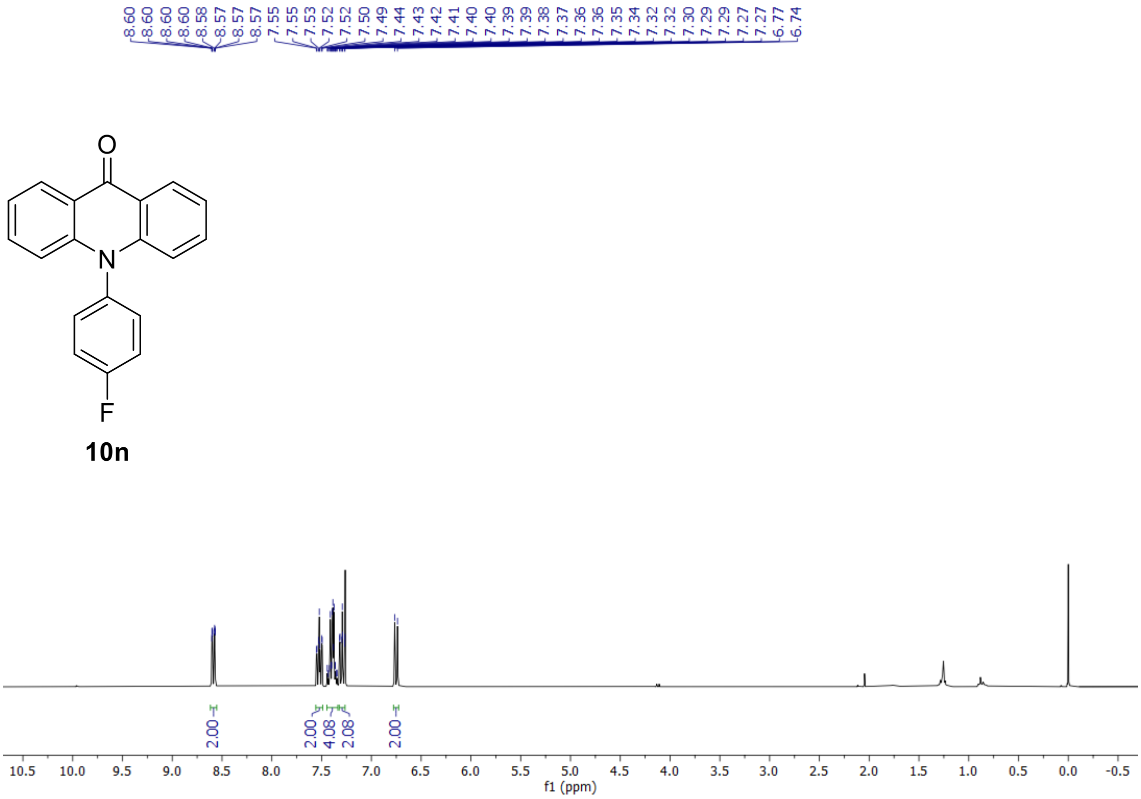

Figure S27. ^{1}H NMR spectrum ($CDCl_3$, 300 MHz) of compound **10n**.

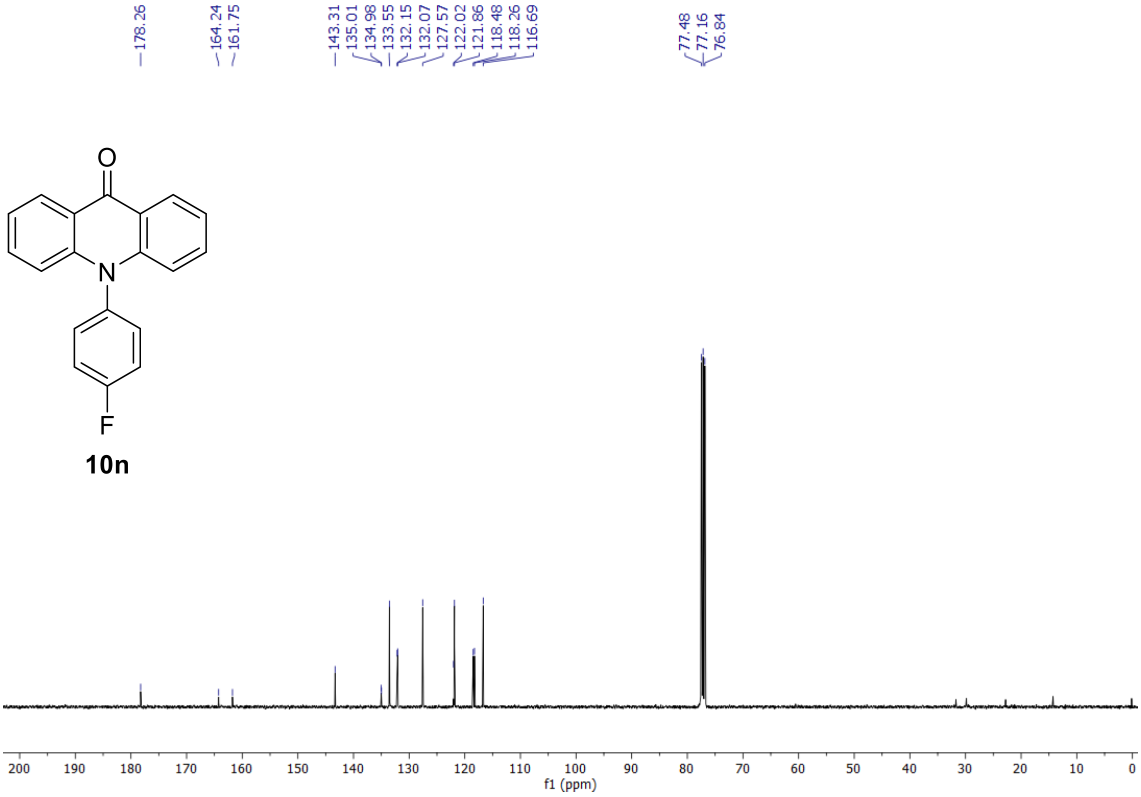

Figure S28. ^{13}C NMR spectrum ($CDCl_3$, 101 MHz) of compound **10n**.

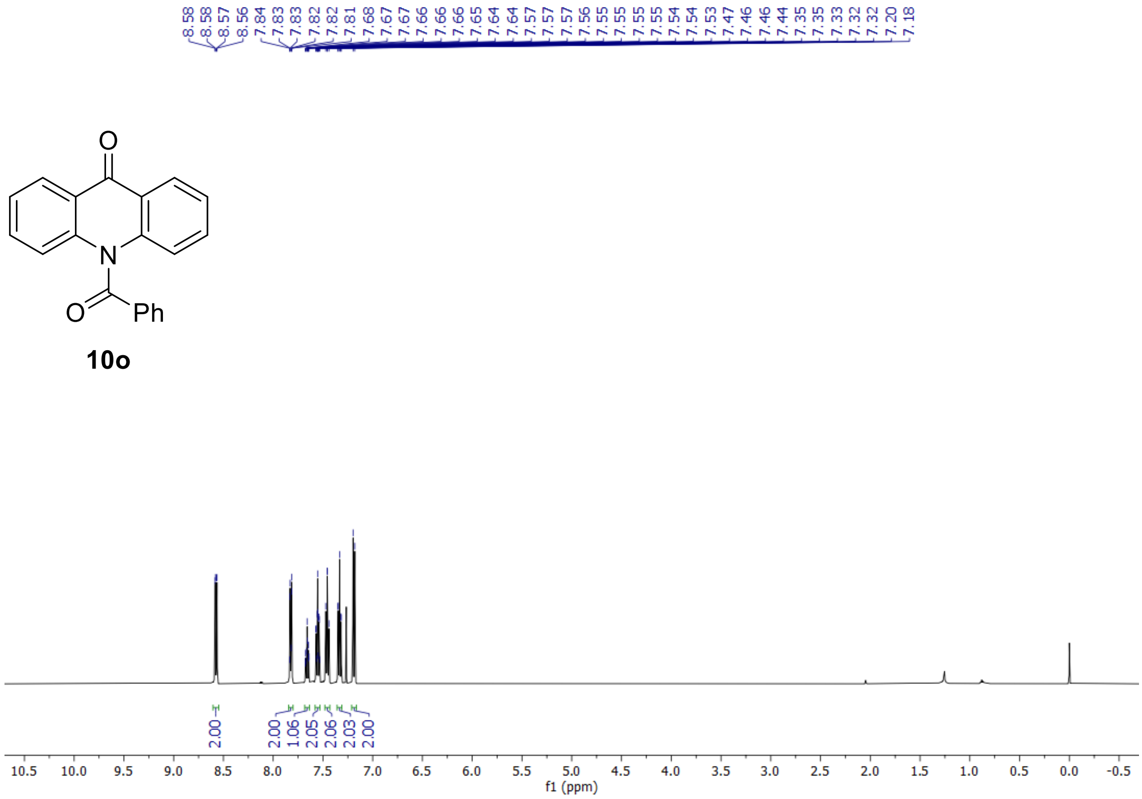

Figure S29. ^{1}H NMR spectrum ($CDCl_3$, 500 MHz) of compound **10o**.

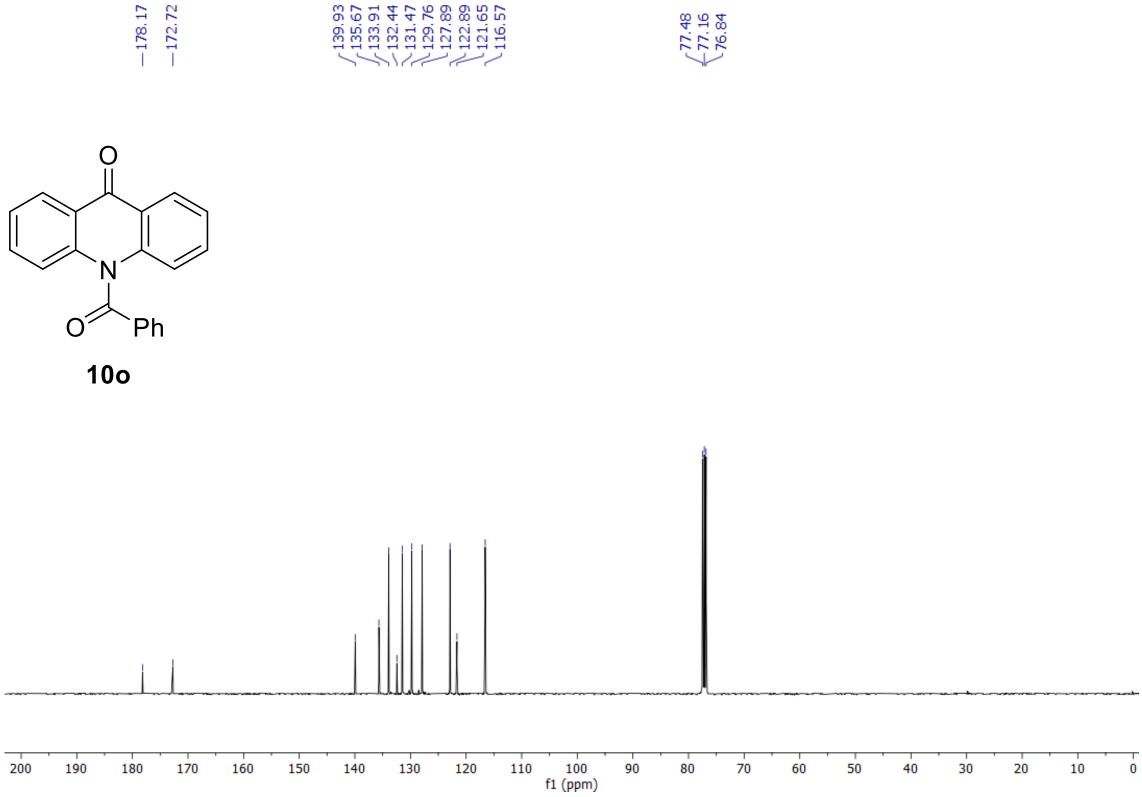

Figure S30. ^{13}C NMR spectrum ($CDCl_3$, 101 MHz) of compound **10o**.

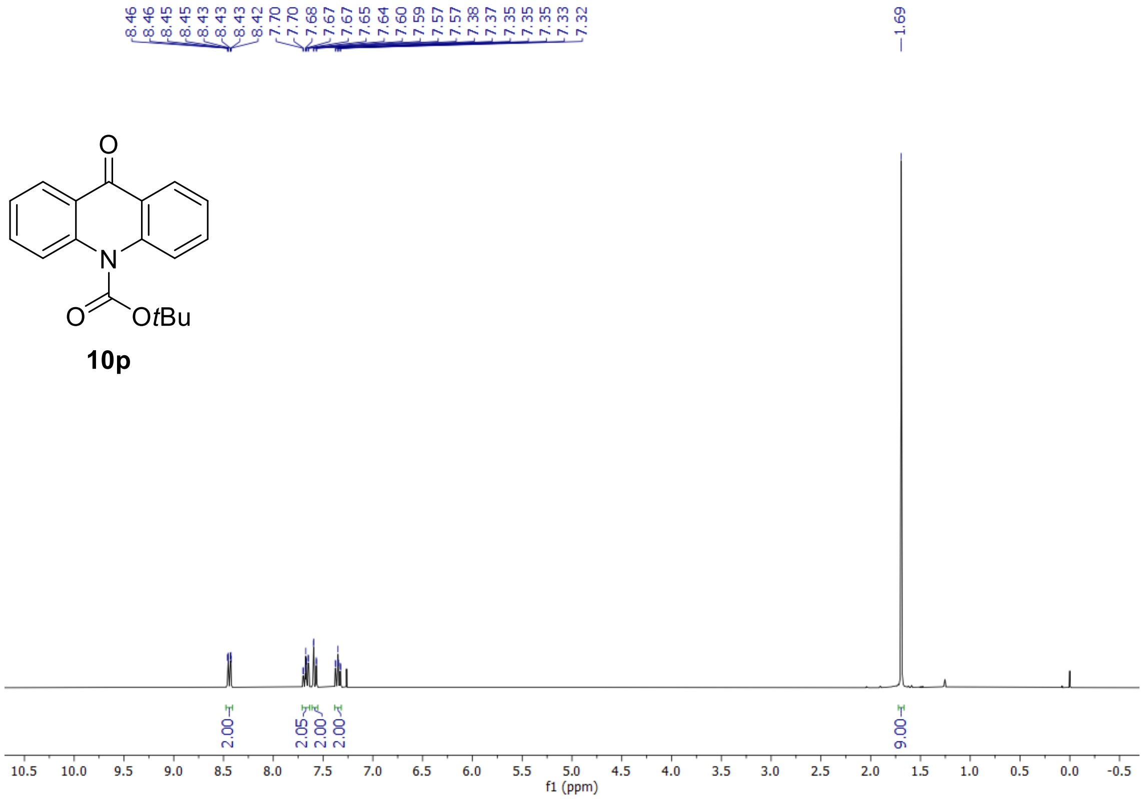

Figure S31. ^{1}H NMR spectrum ($CDCl_3$, 300 MHz) of compound **10p**.

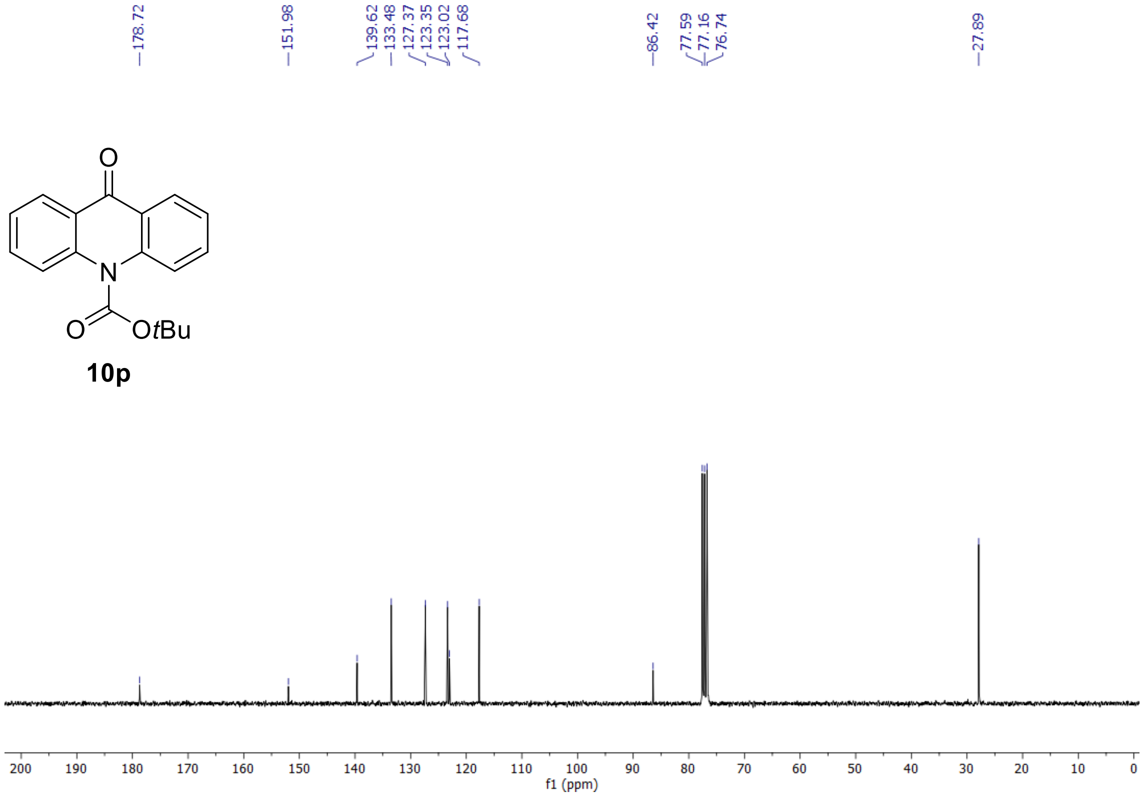

Figure S32. ^{13}C NMR spectrum ($CDCl_3$, 75 MHz) of compound **10p**.

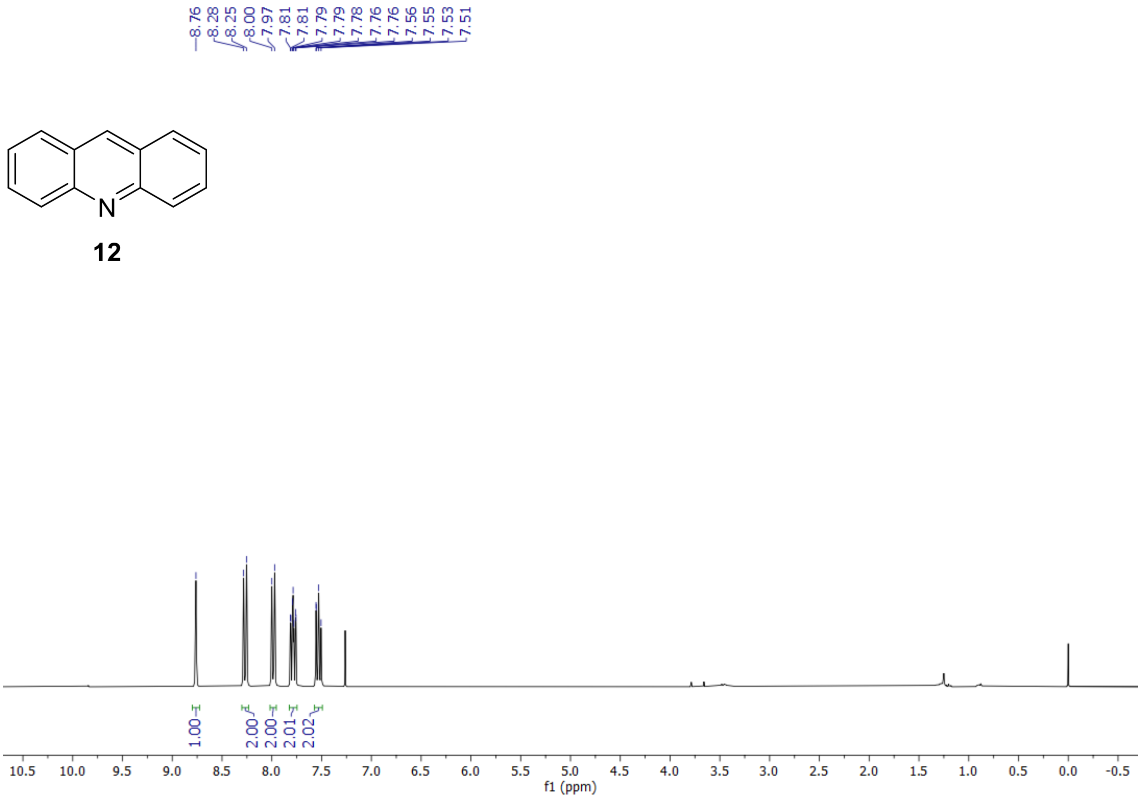

Figure S33. ^{1}H NMR spectrum ($CDCl_3$, 300 MHz) of compound **12**.

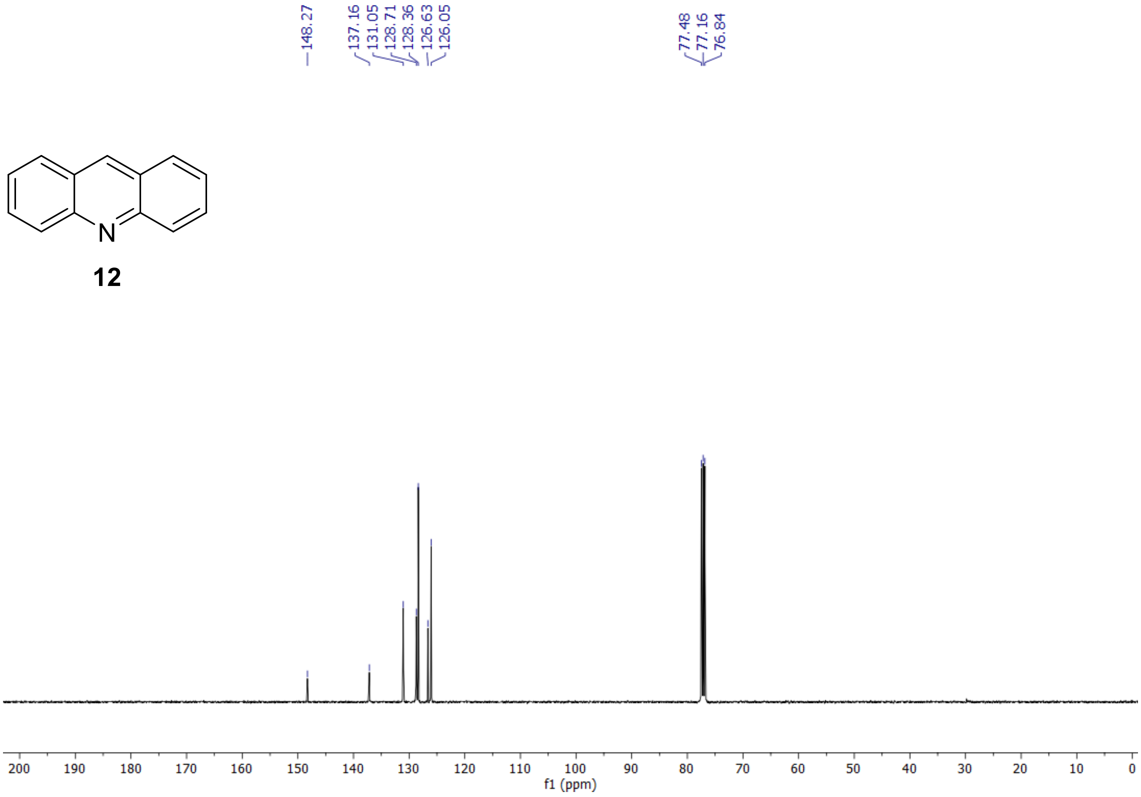

Figure S34. ^{13}C NMR spectrum ($CDCl_3$, 101 MHz) of compound **12**.

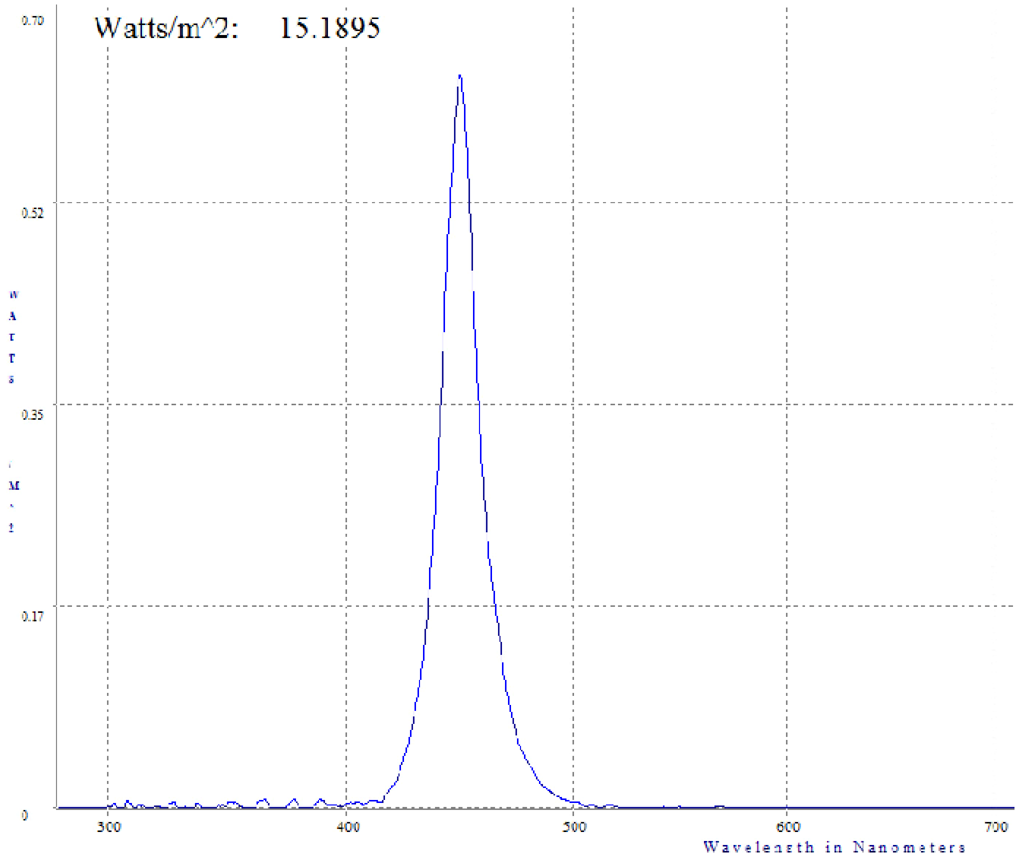

Figure S35. Emission spectrum of the Evoluchem™ CREE XPE 450-455 nm 18W LED (HCK1012-01-002).

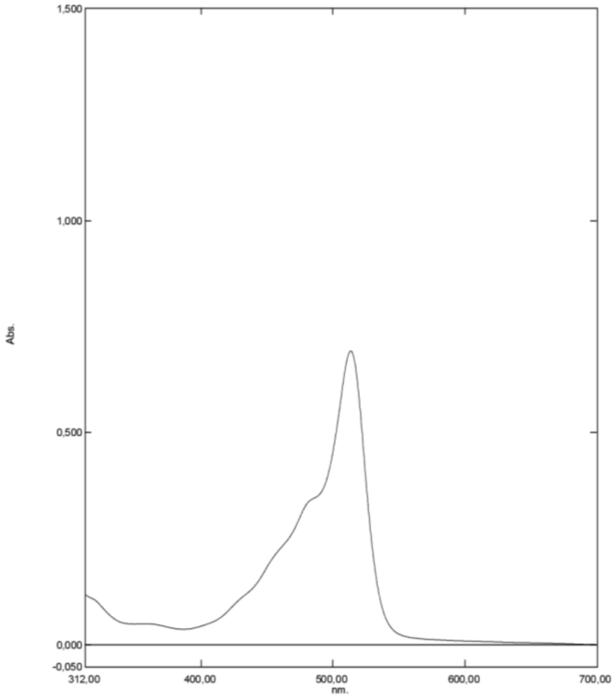

Figure S36. Absorbance UV-Vis spectrum of compound **1** (MeCN, $6.7 \cdot 10^{-4}$M).

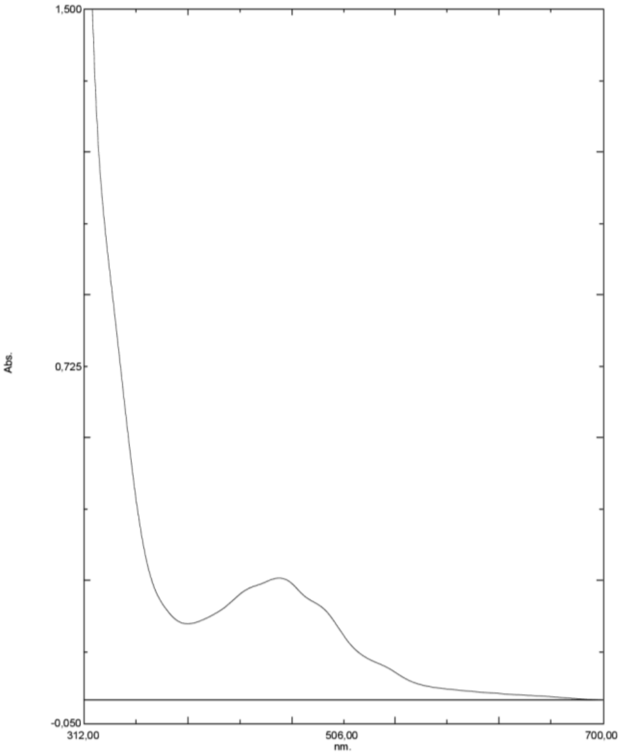

Figure S37. Absorbance UV-Vis spectrum of compound **2** (MeCN, $6.7 \cdot 10^{-4}$M).

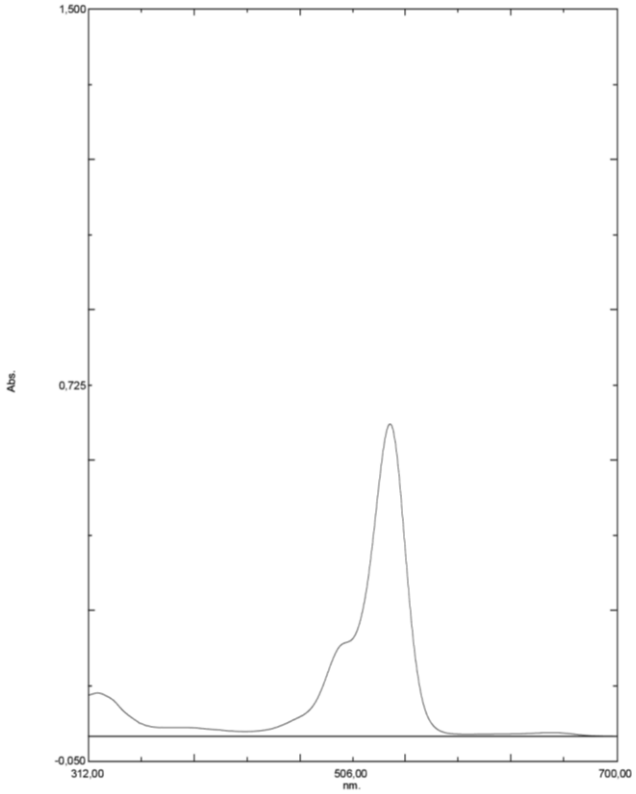

Figure S38. Absorbance UV-Vis spectrum of compound **3** (MeCN, 2.5·10^{-5}M).

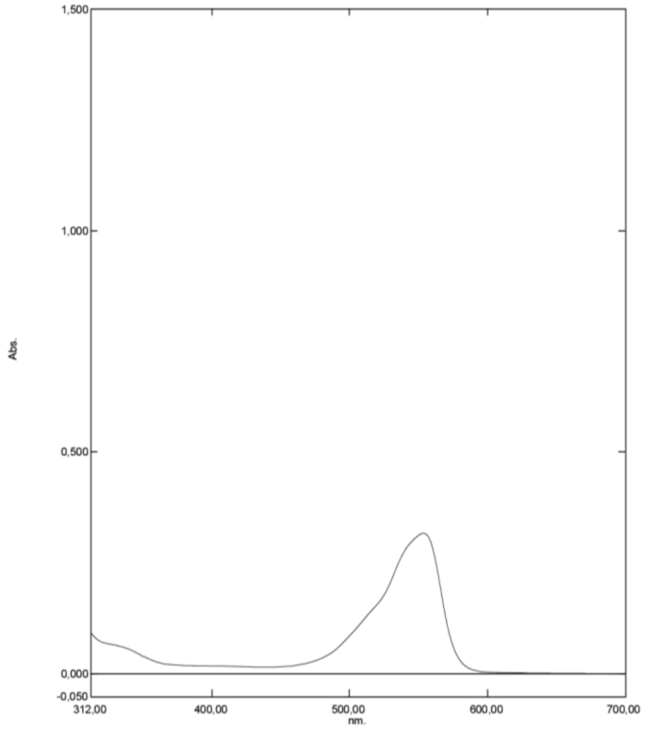

Figure S39. Absorbance UV-Vis spectrum of compound **4** (MeCN, 2.5·10^{-5}M).

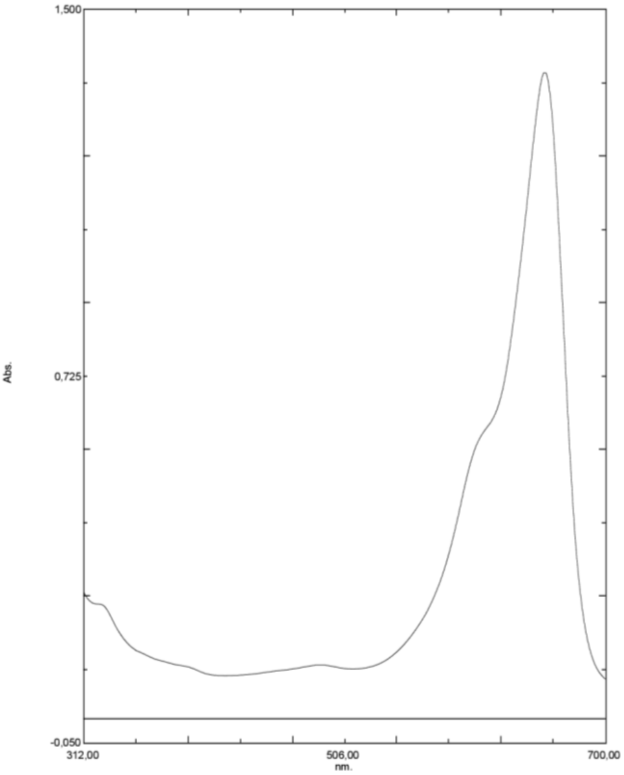

Figure S40. Absorbance UV-Vis spectrum of compound **5** (MeCN, 2.5·10^{-5}M).

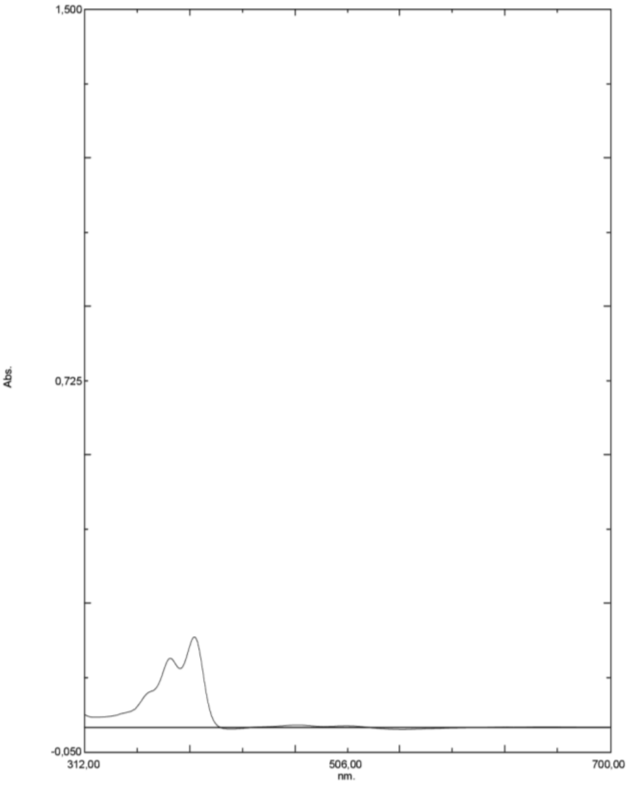

Figure S41. Absorbance UV-Vis spectrum of compound **6** (MeCN, 2.5·10^{-5}M).

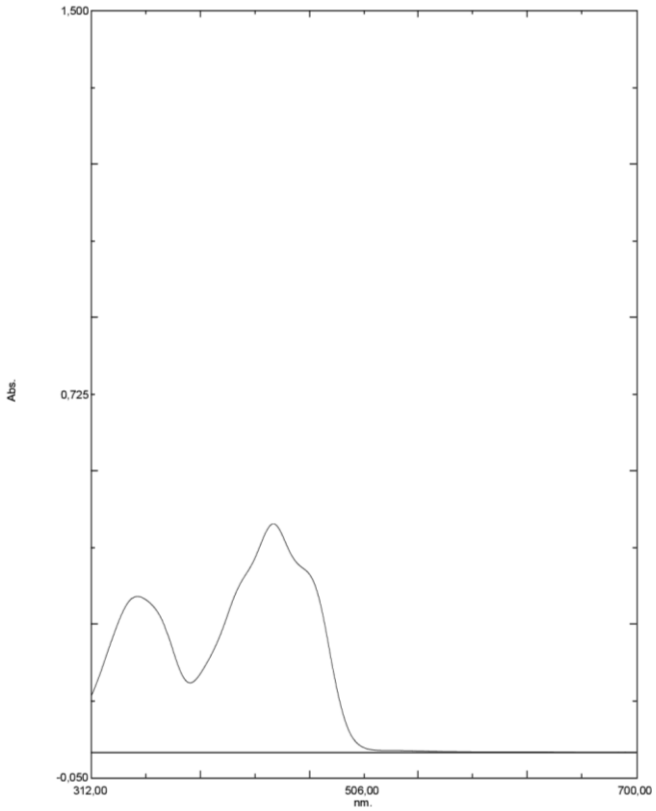

Figure S42. Absorbance UV-Vis spectrum of compound **7** (MeCN, 2.5·10^{-5}M).

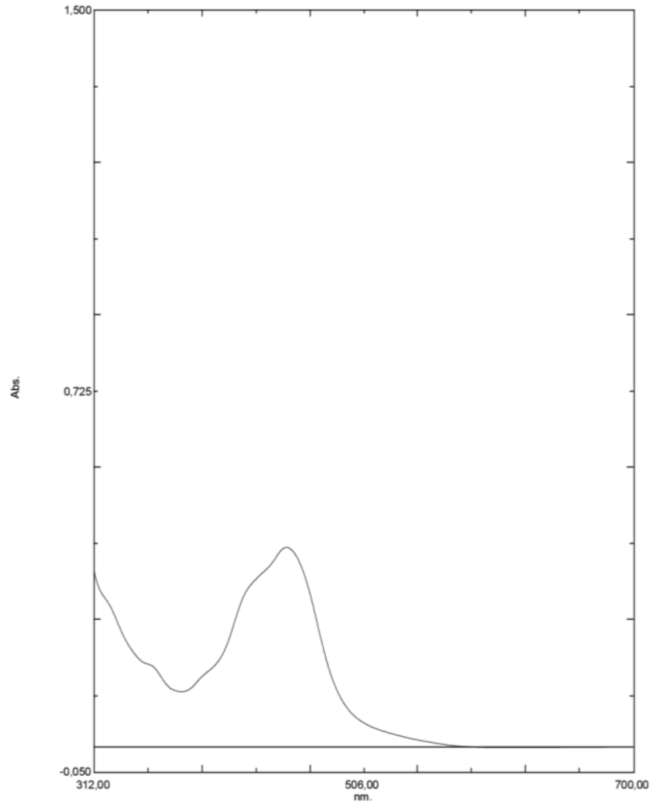

Figure S43. Absorbance UV-Vis spectrum of compound **8** (MeCN, 2.5·10^{-5}M).
